# RNA motif search with data-driven element ordering

**DOI:** 10.1186/s12859-016-1074-x

**Published:** 2016-05-18

**Authors:** Ladislav Rampášek, Randi M. Jimenez, Andrej Lupták, Tomáš Vinař, Broňa Brejová

**Affiliations:** Department of Computer Science, University of Toronto, Toronto, M5R 3G4 ON Canada; Department of Pharmaceutical Sciences, Chemistry, and Molecular Biology and Biochemistry, University of California, Irvine, 2141 Natural Sciences 2, Irvine, 92697 CA USA; Faculty of Mathematics, Physics, and Informatics, Comenius University, Mlynská dolina, Bratislava, 842 48 Slovakia

**Keywords:** RNA motif search, Pseudoknot, Search order, Entropy

## Abstract

**Background:**

In this paper, we study the problem of RNA motif search in long genomic sequences. This approach uses a combination of sequence and structure constraints to uncover new distant homologs of known functional RNAs. The problem is NP-hard and is traditionally solved by backtracking algorithms.

**Results:**

We have designed a new algorithm for RNA motif search and implemented a new motif search tool RNArobo. The tool enhances the RNAbob descriptor language, allowing insertions in helices, which enables better characterization of ribozymes and aptamers. A typical RNA motif consists of multiple elements and the running time of the algorithm is highly dependent on their ordering. By approaching the element ordering problem in a principled way, we demonstrate more than 100-fold speedup of the search for complex motifs compared to previously published tools.

**Conclusions:**

We have developed a new method for RNA motif search that allows for a significant speedup of the search of complex motifs that include pseudoknots. Such speed improvements are crucial at a time when the rate of DNA sequencing outpaces growth in computing. RNArobo is available at http://compbio.fmph.uniba.sk/rnarobo.

**Electronic supplementary material:**

The online version of this article (doi:10.1186/s12859-016-1074-x) contains supplementary material, which is available to authorized users.

## Background

Functional RNAs are often more conserved in their structure than in sequence. Thus to find RNAs related to a known example, we look for sequences capable of assuming the appropriate secondary structure. Here, we investigate the problem of RNA motif search based on user-defined descriptors. RNA motif descriptors specify restrictions on base-pairing structure of the target RNA, as well as sequence constraints characterizing conserved functional sites. As opposed to popular fully-automated systems based on probabilistic models [1–3], this approach allows expert users to handcraft motif descriptors and highlight the most important features of the target RNAs, thus better targeting a particular biological phenomenon [4–7]. In this paper, we revisit the problem of descriptor-based search and present a new tool, RNArobo, that improves the speed of such searches compared to previous methods, including RNAbob [5], RNAMotif [8], RNAMot [4] and RaligNAtor [9].

Currently, most popular tools for RNA motif search, such as InferRNAl [3] or CMFinder [2], are not based on handcrafted motif descriptors. Instead, they use covariance models founded on stochastic context-free grammars, that are built automatically from a set of known occurrences of the target RNA. This approach addresses many shortcomings of descriptor-based methods, most notably the difficulties in deciding which parts of the motif are important for recognizing a particular RNA, as well as high false positive rates of less specific descriptors.

Covariance models are relatively rich probabilistic models, and consequently many examples are required to build a model of a given RNA family. This precondition can be sometimes easily satisfied, most notably in cases where an alignment of the target family is already present in a database, such as Rfam [10].

However, if only a few examples are known for a particular RNA motif, we are under the necessity to find more occurrences before such parameter-rich models can be employed. In such cases, motif descriptors have been used with great success to uncover the distribution of small structured RNAs in the genomic space. These functional RNAs include hammerhead ribozymes [11–17], hepatitis delta virus(HDV)-like ribozymes [7, 18–20], as well as genomic aptamers, including the first known human aptamers [21].

This approach is particularly useful for searching for structures that are hard to predict from simple thermodynamic models, such as pseudoknots and nested multi-pseudoknots. HDV-like ribozymes, which have only five conserved, non-contiguous nucleotides out of approximately 50 necessary to form the minimal catalytically-proficient double-pseudoknot [18, 20], represent a particularly striking example of a functional RNA with low sequence conservation and strict structural requirements. Loose descriptors with low sequence requirements tend to yield large numbers of matches in low-complexity genomic sequences (such as long AT and GT repeats); on the other hand, overly strict descriptors often yield too few or no examples of the sought-for functional RNA. To maximize the yield of bona fide examples of functional RNAs with low sequence requirements, their motif descriptors require careful tuning and multiple runs through available genomic sequences. There is thus a great need for efficient descriptor-based search algorithms.

The specific search problem addressed by our method is NP-hard [22]; hardness was also proved for other similar problems involving alignment with arbitrary non-nested interactions [23, 24]. On the other hand, structures without pseudoknots or with simple pseudoknot configurations can be solved by dynamic programming in polynomial time [6, 24, 25], but the running time is at least cubic in the size of the sequence. Nevertheless, even algorithms with worst-case exponential time were shown to be effective in practice, such as backtracking algorithm of RNAMot [4] or non-deterministic finite-state automata with node rewriting of RNAbob [5]. Many other tools were subsequently developed, including Locomotif [6], Palingol [26], RNAMotif [8], PatSearch [27], and RNAMST [28]. Individual tools differ in descriptor capabilities and post-processing options; an extensive review can be found in [29].

To speed up the search, some tools use advanced data structures to build an index of target DNA. For example, Structator [30] and RNAPattMatch [31] use affix arrays [32] and RaligNAtor [9] uses enhanced suffix arrays [33].

Here, we present a new tool, RNArobo, which builds on the descriptor format of RNAbob and the backtracking algorithm of RNAMot. We improve these tools in two ways. First, we extend the RNAbob descriptor format to allow insertions representing bulges in helical elements. In our experiments, we demonstrate that this seemingly minor change helps to better characterize certain families of ribozymes and aptamers and even enables discovery of new occurrences of these motifs that are likely biologically active. Second, we developed a new method for improving the running time of the backtracking algorithm, in some cases speeding up motif searches more than 100-fold compared to other tools.

Each RNA structure descriptor consists of several structural elements. In our algorithm, individual elements are aligned to the DNA sequence by dynamic programming, with backtracking guiding the search for successive elements to appropriate locations with respect to the already matched elements.

The performance of backtracking depends greatly on ordering of elements in the search. Ideally, the first elements will have few matches, filtering out most of the sequence from further processing. Such filtering is a common theme in many text search methods, such as the popular sequence similarity search tool BLAST [34]. Finding the best element ordering for the backtracking search is an interesting and non-trivial problem, due to complex dependencies between locations of individual elements. We approach this as an on-line problem, using the observed performance of the search so far to adjust the ordering on-the-fly. We demonstrate that this strategy leads to a significant reduction in the running time on real data, especially for complex descriptors.

The rest of the paper is organized as follows. First, we define descriptors and their capabilities, and describe the basic backtracking algorithm. Then we introduce our data-driven element ordering strategy. Finally, we demonstrate effectiveness of our approach by revisiting the results of several biological studies and compare our running time with several existing tools. The software tool RNArobo implementing improvements described in this paper is available at http://compbio.fmph.uniba.sk/rnarobo.

## Methods

### Descriptor-based search for RNA motifs

Here, we briefly describe the search algorithm implemented in our tool RNArobo which is loosely based on the algorithm of RNAMot [4]. The input for the algorithm is a *descriptor* specifying the desired RNA structural motif and a DNA sequence. The goal is to find all occurrences of the motif in the sequence. A descriptor consists of three parts:

1. a *motif map* – a list of individual *structural elements* ordered from 5’ to 3’ end along the sequence,
2. a detailed *specification* of each structural element,
3. an optional *search order*.

**Fig. 1:**
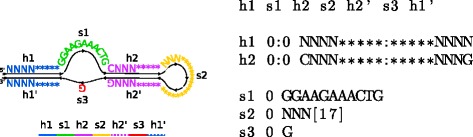
An illustration of an ATP aptamer motif and its corresponding descriptor based on genomic adenosine aptamers [21]. Nucleotide constraints for individual positions are expressed in the IUPAC notation [51]

Each structural element is either single-stranded or paired (helical). *Single-stranded elements* are regular expressions, similar to those used in PROSITE [35]. The user can also allow a fixed number of mismatches and insertions to appear anywhere in the motif. *Paired elements* correspond to helices in the RNA structure and consist of two interacting regions of the DNA sequence. The descriptor can specify the minimum and maximum length of the helix, sequence constraints in the form of a regular expression, as well as constraints on the paired bases (for example, we can consider only canonical Watson-Crick base-pairs, or allow U-G pairs as well). Again, users can allow a certain number of mispairs between paired bases, mismatches with respect to the sequence constraints, and insertions of single-base bulges. Each paired element occurs twice in the motif map, specifying the location of both strands. We place no restrictions on the relative order of elements in the motif map, and thus the descriptor can specify arbitrary pseudoknotted structures. An example of a descriptor is in Fig. 1, and the full description of the file format is given in Additional file 1: Section S1.

The user can optionally specify the search order in which individual elements will be considered in the backtracking search. The search order has a large influence on the running time, and the main focus of this paper is automated selection of appropriate search orders.

**Fig. 2:**
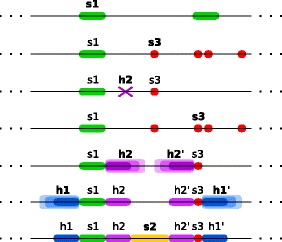
An illustration of the RNArobo search procedure for the motif of ATP aptamer. The search follows the order of elements s1, s3, h2, h1, s2

**Algorithm outline** The algorithm uses a simple backtracking strategy with a fixed search order of elements *e*_1_,*e*_2_,…,*e*_*n*_. First, we find all matches of element *e*_1_ in a certain sequence window *T*. Then we consider each match of *e*_1_ in turn and try to expand it to an occurrence of the complete motif by recursively searching for matches of *e*_2_,…,*e*_*n*_ in appropriate relative positions with respect to the match of *e*_1_. An illustration of the search procedure is depicted in Fig. 2.

To find matches of element *e*_*i*_, we devised general but relatively slow dynamic programming algorithms. For single-strand elements with no wild cards or insertions, we use a much faster bit-parallel bounded nondeterministic DAWG matching algorithm [36]. For the rest of the single-strand elements, we first use bit-parallel shift-and forward filtering [37] to identify sequence positions with possible element occurrences, and only subsequently we verify matches by the full dynamic programming algorithm.

The dynamic programming tables of our algorithms have many dimensions, because we need to keep track of the number of insertions, mismatches, and for paired elements also mispairs. In a typical sequence search scenario, each of these differences is assigned some negative score and the goal is to optimize the overall score. In contrast, we have a separate upper bound for each type of difference from the motif; therefore, each type adds another dimension to the dynamic programming table.

For example, for paired elements, our table *H* has seven dimensions. The value of $H_{t_{1},t_{2},p,m,r,i,b}$ is TRUE if and only if prefix *P*[1…*p*] of the regular expression can be aligned with a suffix *T*^′^ of *T*[1…*t*_1_] with *m* mismatches, a prefix *P*^′^[1…*p*] of the regular expression for the reverse strand can be aligned with a prefix *T*^″^ of *T*[*t*_2_…|*T*|] with no mismatches, and the alignment of *T*^′^ and *T*^″^ to each other contains *i* insertions and *r* mispairings. Furthermore, since we do not allow insertions to be adjacent, we use a binary flag *b* such that *b* is true if and only if one of *T*[*t*_1_] and *T*[*t*_2_] is an insertion. The complete dynamic programming recurrences can be found in Additional file 1: Section S2.

The number of allowed insertions, mismatches, and mispairs is typically very small, and thus the dynamic programming runs in *O*(*t*^2^*k*) time, where *t* is the length of the sequence window *T* and *k* is the length of the motif. The search procedure divides the whole sequence into windows of size max{20*L*,3000}, where *L* is the maximum length of an occurrence. Successive windows overlap by length *L* so that each occurrence is guaranteed to be completely contained in at least one window.

**Fig. 3:**
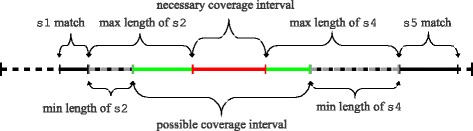
Computation of the search domain for a single-stranded element s3. Here we have a partly matched motif composed of five single-stranded elements s1,…,s5. Assume that elements s1 and s5 have already been matched. The match of s3 has to start in the left green interval and end in the right green interval, and it has to completely cover the red interval in the middle

For the first element *e*_1_ in the search order, we run the search on the whole window. For the successive elements, we compute a *search domain* in which this element may occur and restrict the dynamic programming accordingly. The search domain is determined based on the positions of the closest matches on the left and on the right already fixed in the previous steps of the backtracking search and by the flexibility in the length of the elements separating the matches of previously fixed elements from the current element, as illustrated in Fig. 3.

The overall running time of our algorithm can be, in the worst case, exponential in the number of elements of the descriptor. However, the number of these elements is typically small, and if we use a well-chosen search order, the early branching elements will have relatively few matches, thus limiting the degree of the search tree. Many branches of the search are terminated early, because no match of an element is found in its search domain.

### Element ordering

The search order of elements significantly affects the running time. In this section, we present our data-driven element ordering (DDEO) strategy. In general, it is advantageous to start with elements that have few matches, thus eliminating a large portion of the sequence to be searched. Once the matches of some elements are found, it is also important to consider flexibility of the placement of a new element with respect to those that are already matched.

While these principles are quite natural, it is difficult to transform them into an effective criterion for creating good search orders. Therefore, we propose a data-driven method for finding a close-to-optimal element ordering.

Our approach consists of two parts. First, we use a heuristic approach to create a set *O* of candidate orderings. We then use these orderings on sequence windows, measure their actual performance, and select the best one. We do this while processing the initial segment of the query sequence, thus limiting the amount of overhead spent on selecting a suitable search order.

#### Heuristic proposal of element orders

To create the proposal set *O*, we concentrate on the first *k*-tuple of elements in the search order (in experiments, we use *k*=3). We create all possible *k*-tuples and score them by the heuristic scoring function described below. All the *k*-tuples with scores above some threshold are then augmented to complete search orders forming the proposal set *O*. In experiments we select *k*-tuples that achieve score that is at least 85 *%* of the maximum among all *k*-tuple scores. We limit the size of *O* to 50 if there are too many good candidates.

The goal of this initial heuristic evaluation is to select a small subset of *k*-tuples to be evaluated empirically. We want this subset to include *k*-tuples that can be augmented to complete search orders yielding running times close to the optimum. Conversely, we should not include too many tuples yielding slow running times, because their evaluation will increase the overall running time.

The score of a *k*-tuple *e*_1_,…,*e*_*k*_ is a weighted sum of two heuristic functions evaluated for individual elements

1 $$ {}\begin{aligned} h(e_{1},\dots, e_{k}) = \sum_{i=1}^{k} 2^{k-i} \left(c_{1} \cdot h_{1}(e_{i}) + c_{2} \cdot h_{2}(e_{1}, \dots, e_{i}) \right) \end{aligned}  $$

Function *h*_1_(*e*_*i*_) approximates the information content of element *e*_*i*_ and function *h*_2_(*e*_1_,…,*e*_*i*_) considers flexibility of element *e*_*i*_ with respect to the already matched elements *e*_1_,…,*e*_*i*−1_. Note that element weights decrease exponentially, because elements placed earlier in the search order tend to be searched in a larger portion of the sequence. We set the weight *c*_2_ in the linear function to −0.2, while *c*_1_ is 1 for paired elements and 3 for single-strand elements to reflect that the search for unpaired elements is considerably faster.

#### Information content heuristic

The first heuristic function *h*_1_ is an approximation of the information content of an element, favoring elements that pose more specific constraints. Thus this function follows the *fail-first* rule generally used in backtracking searches [38]. *Information content* is a measure of uncertainty reduction about an outcome once we have received a new piece of information. In particular, it is the difference in the entropy of a random variable before and after some message has been received. The entropy of a discrete random variable *X* with possible values *x*_1_,*x*_2_,… is defined as

2 $$ H(X) = -\sum_{i} P[X=x_{i}]\log_{2} {P[X=x_{i}]}.  $$

Let us first consider a single-stranded element *S*, and let *N* be the longest possible occurrence of this element. In our setting, the random variable is a sequence of length *N* and the message is that the sequence starts with a match of the pattern. To estimate the background entropy before receiving the message, we consider all 4^*N*^ sequences of length *N* equally likely, obtaining

3 $$ H_{\text{before}} = -\sum_{i=1}^{4^{N}} \frac{1}{4^{N}} \log_{2}{\frac{1}{4^{N}}} = 2N.  $$

We compare this value with the entropy of the uniform distribution over all sequences of length *N* that have an occurrence of the element starting at the first position. If *X* is the number of such sequences, we have *H*_after_= log2*X* and the information content of *S* is

4 $$\begin{array}{*{20}l} h_{1}(S) = H_{\text{before}} - H_{\text{after}} = 2N - \log_{2}{X}. \end{array} $$

Since the value of *X* is hard to compute for complex elements, we use an upper bound *X*_*U*_≥*X* (which leads to a lower bound for the information content of *S*). To obtain the upper bound *X*_*U*_, we count different ways of obtaining a sequence matching *S*, disregarding the fact that some sequences may be obtained in several different ways and consequently counted multiple times.

In the simplest case, element *S* does not contain any flexible-length wild cards and does not allow for any distortions (mismatches, insertions). The element specifies for each position *i* the set of allowed nucleotides; let *C*[*i*] be the size of this set. The value of *X* is then simply

5 $$ X = \prod_{i=1}^{N} C[i].   $$

Next we extend the bound to cases when *S* contains wild cards. Each wild card corresponds to an arbitrary nucleotide or to an empty string. A block of *k* consecutive wild cards thus corresponds to an arbitrary sequence of length up to *k*. Let *X*_1_ be the value obtained by formula (5) if we consider only non-wild card positions in *S*. A single block of *k* consecutive wild cards increases the value of *N* (the length of the longest occurrence of *S*) by *k*. These *k* additional nucleotides can be arbitrary, and are split into a block of length *i* matching the block of wild cards and a block of length *k*−*i* located after the element occurrence (this block corresponds to the unused wild cards). Since the value of *i* can be any integer between 0 and *k*, this leads to the upper bound of *X*_1_(*k*+1)4^*k*^ sequences matching *S*. If *S* has multiple blocks of wild cards of lengths *k*_1_,…,*k*_*b*_, each of them can be split into two blocks independently, leading to the upper bound

6 $$\begin{array}{*{20}l} X_{2} = X_{1} \cdot \prod_{i=1}^{b} 4^{k_{i}}(k_{i}+1). \end{array} $$

Similarly, we adjust the value of *X* to account for mismatches and insertions allowed in the element to obtain the final upper bound *X*_*U*_; see details in Additional file 1: Section S3. For practical reasons, we handle mismatches using a formula which is not guaranteed to be an upper bound of the real set size *X* for each motif, but works well in practice.

The situation is analogous for paired elements. Let *H* be an element consisting of two paired strands *H*_1_ and *H*_2_, and let *N* be the maximum length of a match to one of these two strands, after accounting for wild cards and insertions. Since we now consider sequences of total length 2*N*, the background entropy is

$$ H_{\text{before}} = \log_{2} 4^{2N} = 4N. $$

We use *H*_after_= log2*X*, where *X* is the number of pairs of sequences of length *N* such that *H*_1_ occurs in the first sequence starting at the first position, and *H*_2_ occurs in the second sequence ending at the last position, and these two occurrences satisfy the complementarity constraints with up to allowed amount of distortion. We again use an approximate upper bound *X*_*U*_ instead of the actual count *X*, counting different ways that such a matching can occur.

As with single-stranded elements, we first count the number of sequences that match *H* without considering wild cards and distortions. Let *P*[*i*] be the number of valid base pairs between position *i* of *H*_1_ and the corresponding position of *H*_2_. The value of *P*[*i*] is determined by both complementarity constraints specified by *H* and by sequence constraints for the respective positions in *H*_1_ and *H*_2_. As before, the number of matching sequences is the product

7 $$\begin{array}{*{20}l} X_{1} = \prod_{i=1}^{N} P[i]. \end{array} $$

To obtain the final bound *X*_*U*_, we adjust the value of *X*_1_ to account for wild cards, mismatches, and insertions, similarly as in the single-stranded case. We also adjust the bound for allowed mispairs, where the two paired nucleotides do not form a valid base pair. Details can be found in Additional file 1: Section S3.

#### Domain flexibility heuristic

The second heuristic scoring function *h*_2_(*e*_*i*_) measures the flexibility of positioning element *e*_*i*_ with respect to already matched elements *e*_1_,…,*e*_*i*−1_. The matches of these elements specify the search domain for element *e*_*i*_, as shown in Fig. 3. Longer domains require more time for finding matches and are also likely to yield more matches, each of which will be then examined individually in backtracking. Therefore, we set the weight *c* in (1) to be negative.

To compute the exact size of the search domain, we need to know positions of matches of *e*_1_,…,*e*_*i*−1_. In order to score a particular search order before the search starts, we need to approximate flexibility of *e*_*i*_ without this knowledge. For an unpaired element *e*_*i*_, we find the nearest fixed element *e*_*ℓ*_ on the left side of *e*_*i*_ (one of *e*_1_,…,*e*_*i*−1_). Then we sum up the flexibilities of all elements between *e*_*ℓ*_ and *e*_*i*_ in the descriptor, where the flexibility of an element is the difference between its maximum and minimum length. We denote this sum *F*_left_. Analogously, we obtain *F*_right_ for the right side of *e*_*i*_. If there is a fixed element on both sides of *e*_*i*_, we take the minimum of *F*_left_ and *F*_right_, as both are upper bounds on the search domain size for *e*_*i*_:

8 $$ h_{2}(e_{1}, \dots, e_{i}) = \min \{F_{\text{left}}, F_{\text{right}} \}.  $$

If there is no fixed element at one side, we only use the other side to compute *h*_2_. For a paired element *e*_*i*_, we first compute flexibilities of the two strands individually, while considering the other strand to be fixed. Then we take the maximum of these two.

#### Candidate order elimination

The heuristic function defined above is used to select promising initial *k*-tuples for search orders. These are then completed to full search orders by adding remaining elements in the order determined by the information content heuristic, forming the initial candidate set *O*.

The initial candidate set contains a mix of good and bad orderings. We process several window s of the sequence to determine which orderings are good. For each window, we sample a random search order *x* from *O* and use it in the search procedure, measuring its performance *T*_*x*_. In particular, we record nanoseconds used by the process (measured by an available system function) and normalize it by the window size. Based on the gathered data, we continually eliminate orderings with bad performance using a statistical test.

We treat *T*_*x*_ as a random variable, and approximate it by the normal distribution with an unknown mean and variance. Our goal is to pick from the set of candidate orderings *O* the ordering leading to the shortest mean execution time. Formally, we want to find *x*^∗^∈*O*, such that for each *y*, $\phantom {\dot {i}\!}E[T_{x^{*}}] \leq E[T_{y}]$. Given several observed values of *T*_*x*_ and *T*_*y*_, we use Welch’s t-test [39] to test the null hypothesis *E*[*T*_*x*_]≥*E*[*T*_*y*_] against the alternative hypothesis *E*[*T*_*x*_]<*E*[*T*_*y*_]. This test is used for hypothesis testing concerning difference between the actual means of two normally distributed populations with possibly unequal variances, based on independent sets of samples from these distributions [40].

Each time we gather a new sample from *T*_*x*_ for some *x*∈*O*, we test *x* against the rest of *O*. When we observe a statistically significant difference between two candidates (at the level *α*=0.01), we eliminate the one with the higher mean time of execution from set *O*. If two candidates cannot be shown significantly different, even after both were sampled many times (75 samples from each), we simply eliminate the ordering with the higher sample mean.

Once we eliminate all but one search order from set *O*, we start to refine this final ordering. Recall that it consists of an initial *k*-tuple extended to a full ordering by the information content heuristic. We drop this heuristic extension, and start training the following *k*-tuple in the search order with the first *k*-tuple already fixed. We continue until we completely fix the search order.

#### Performance of DDEO

**Fig. 4:**
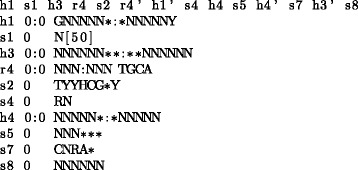
Descriptor for HDV-like ribozyme with structured P4 region. The motif contains four paired elements organized in a double pseudoknot

We have evaluated DDEO heuristic on the hepatitis delta virus like ribozyme (HDV) descriptor (Fig. 4). Even though the entropy-based heuristic is not perfect and the candidate set contains bad orderings in addition to the good ones, the orderings with bad performance are eliminated after only a few samples (in some cases as few as two). These “bad runs” thus do not increase the overall running time significantly. (More details can be found in Additional file 1: Section S4).

## Results and Discussion

We have performed several experiments comparing RNArobo with other established RNA motif search tools: RNAbob ([5], software version 2.2.1 from 2012), RNAmotif ([8], software version 3.0.7 from 2010), RNAMot ([41], software version 2.1 from 1994), and RaligNAtor [9]. We have concentrated on both the speed and the ability to discover biologically meaningful motif occurrences.

**Accuracy of hits** Table 1 shows the results of an experiment, where we revisited several scientific studies involving discovery of ribozymes and aptamers [7, 16, 42]. We have constructed RNA descriptors for RNAbob and used both RNAbob and our new tool RNArobo to identify motif occurrences. Extended description of the experiment and the descriptors are included in Additional file 1: Section S5. As expected, the results of the two programs were identical, with RNArobo running much faster (data not shown, but see speed evaluation below). Almost all of the hits found by the programs were occurrences of known targets also identified in the original studies, confirming the accuracy of the algorithm (see exceptions below).

**Table 1:** Summary of reproducing results from the literature using RNArobo. Searches were extended by allowing for insertions in structurally conserved elements that are known to tolerate single base insertions. This extension led to improved sensitivity and yielded several new putatively functional ribozymes

*Sequence*	*Descriptor*	*#hits*	*Known targets found*	*Note*
In vitro selected library	GTP apt. class I	9	yes	
(∼65 kBp scanned)	GTP apt. class I w/ ins	10	yes [42]	novel hit
*Yarrowia lipolytica*	HHR I (4 bp)	1	Yli-1-3	
(∼41 MBp scanned)	HHR I (4 bp) w/ ins	15	Yli-1-3 through Yli-1-11	
	HHR I (3 bp)	4	Yli-1-3 and Yli-1-13	
	HHR I (3 bp) w/ ins	54	Yli-1-3 through Yli-1-11,	novel family
			and Yli-1-13 [16]	(10 hits)
*Bacillus cereus*	HHR II	1	Bce-1-1	
(∼11 MBp scanned)	HHR II w/ ins	4	Bce-1-1 [16]	
*Anopheles gambiae* chr2L	HDV (loose P4)	7	Agam-1-1	
(∼98 MBp bases scanned)	HDV (loose P4) w/ ins	36	Agam-1-1 and Agam-1-2 [7]	
*Strongylocentrotus purpuratus*	HDV (stem P4)	11	yes	
(∼2.1 GBp scanned)	HDV (stem P4) w/ ins	11	yes	
	HDV (loose P4) + FF	15	yes	
	HDV (loose P4) w/ ins + FF	16	yes [7]	novel hit

FF: only hits passing the Fold-Filter are reported

**Novel findings** Compared to RNAbob, RNArobo allows insertions (bulges) in helix elements, enabling much more flexible descriptors better characterizing certain families. For example, in the case of hammerhead type I ribozyme (HHR type I, 4 bp), the descriptor with insertions identified 15 known occurrences in *Yarrowia lipolytica* (“Yli” family) [16], compared to a single occurrence identified without insertions.

Allowing insertions, we have also discovered several new candidate occurrences. Firstly, three new hits of hammerhead ribozyme (HHR type II) in *B. cereus* are likely false positives as determined by the Fold-Filter pipeline [43]. This pipeline uses tools from ViennaRNA package [44] and DotKnot [45] to determine if an occurrence of a motif is likely to assume the secondary structure implied by the descriptor.

On the other hand, a novel GTP aptamer (GTP class I) in Davis and Szostak library is likely functional, since the library was selected for GTP binding.

In case of hammerhead ribozyme (HHR type I, 3 bp) in *Y. lipolytica*, the number of hits increased massively from 4 to 54 by allowing insertions. These hits form two distinct families. The first contains previously known “Yli” ribozymes, as identified by Perreault et al. [16]. However, the ten hits of the second family are novel and pass through the Fold-Filter pipeline. They likely represent a novel HHR family in *Y. lipolytica* genome similar to a large family of HHRs in the *Schistosoma mansoni* genome (see also Additional file 1: Figure S7); no HHR type I families besides the “Yli” ribozymes were previously found in *Y. lipolytica*.

Allowing insertions in HDV-like ribozymes in *S. purpuratus* genome did not yield any new hits (HDV stem P4 descriptor). We have attempted to loosen the constraints on the P4 region, which yielded numerous hits both with and without insertions. Consequently, we have applied Fold-Filter and kept only hits passing the pipeline. The descriptor with insertions yielded a previously unidentified hit that aligns well with other HDV ribozymes, thus likely being functional. The above examples show that RNArobo can be helpful in finding interesting novel occurrences, even for known families established in the literature.

**Speed comparison** Table 2 shows the comparison of running times of scanning both strands of the whole human genome for occurrences of nine realistic RNA motifs. In most cases, RNArobo is the fastest tool, in many cases speeding up the search more than 100-fold. Note that running times of both RNAbob and RNAmotif greatly depend on the complexity of the motif, while RNArobo does not show pronounced dependency on the descriptor. This is most apparent for the HDV descriptors which feature a double pseudoknot. Allowing insertions in the helices does not significantly slow down RNArobo search.

**Table 2:** The running times (in seconds) of different programs searching for various descriptors in the whole human genome

	ATP apt.	GTP apt.	generalized	HHR-I	HHR-II	HHR	HDV	HDV	HDV
		class I	tRNA	(4 bp)	(3 bp)	extended	(loose P4)	(stem P4)	(mispairs)
RNAbob	3,419.03	2,744.30	7,450.07	923.53	5,027.33	2,269.35	209,932.57	43,430.78	36,459.87
RNAmotif	**80.78**	222.54	7,374.32	**87.69**	265.09	116.15	26,259.79	2,513.90	9,240.83
RNAMot	unf	unf	unf	unf	unf	unf	unf	4,538.92	8,925.31
RNArobo	80.91	**151.09**	**250.63**	96.48	**110.64**	**108.46**	**171.16**	**169.90**	**111.30**
RNArobo-ins	–	153.38	–	98.22	137.47	–	173.82	171.54	–

Experiments were run on Intel Xeon E5520 CPU. RNArobo-ins is RNArobo run with modified descriptors allowing insertions in helical elements. RNAMot did not finish on most of the inputs within time limit of three days. Only results that finished within three days are shown. Since DDEO is randomized, we show the average running time of five runs of RNArobo. Standard deviation was up to 3 % or 5 sec, with the exception of the HHR extended descriptor, where the running time ranged from 98 to 125 sec

Boldface numbers represent the best running times for a particular descriptor

RaligNAtor [9] is a recent addition to the family of descriptor-based search tools. In contrast to the previous works, the authors add a preprocessing step building index data structures that help to speed up the subsequent searches. Unfortunately, the structural pattern definition language of RaligNAtor is very different from that used by other tools; therefore it is difficult to translate RNArobo patterns to RaligNAtor and vice versa. Nevertheless, we defined approximate counterparts of two patterns (IRES from RaligNAtor tests and generalized tRNA from our tests) in the other descriptor language in order to compare the two tools. Results convincingly show that RNArobo outperforms RaligNAtor in terms of the running time from four-fold to more than 1000-fold (Additional file 1: Table S2). These results hold whether we used exact or approximate patterns, with or without preprocessing (see Additional file 1: Section S7).

**Comparison to convariance-model-based tools** Descriptor-based tools, such as RNArobo, are used by researchers to explore motif families for which only a few occurrences are known, and are heavily based on incorporating user’s intuition in building motif descritors. On the other hand, covariance models, such as InfeRNAl [3], can be used to search for new instances for motif families where many occurrences are already known, and their common features are extracted automatically and encoded in the covariance model. Due to this substantial difference, it is difficult to design a fair comparison between these two classes of algorithms.

Nevertheless, we have attempted to compare RNArobo to InfeRNAl in case of the hammerhead ribozyme family that has been previously extensively studied by several groups. InfeRNAl was generally 3–10 times slower than RNArobo when searching for these motifs in *Yarrowia lipolytica*. Both programs identify bona fide ribozymes; however, InfeRNAl also identifies candidates with mutations in the active site of the catalytic RNA, making these instances most likely inactive. Such results may be useful for RNAs that tolerate a certain level of global mutation rate, but in our case we most likely find false positives. The descriptor of RNArobo allows specification of regions that are under strong purifying selection and need to be conserved; on the other hand, allowing insertions in helices allowed RNArobo to discover a new putative family of *Yarrowia lipolytica* hammerhead ribozymes that InfeRNAl did not find.

## Conclusions

In this work, we have developed a new tool RNArobo for RNA motif search. RNArobo allows expert human users to describe the most relevant features of a target RNA structure and then to search for distant homologs in available genomic data. The focus of our work was an automated strategy for element ordering in the backtracking search employing a heuristic scoring function based on information content and search domain size estimates. We used statistical tests to eliminate candidate orderings that do not perform well in practice. Our experiments demonstrate that RNArobo is much faster for complex motifs than existing tools, thus facilitating large-scale whole-genome searches.

Our work leaves open further avenues for research. The problem of finding the best element ordering, or even estimating the expected number of matches of a single element in a random sequence, is very intriguing from the theoretical point of view. Even though our simple elimination scheme proved to be effective in practice, it would be interesting to treat the problem as an on-line learning problem and develop a theory that would allow us to estimate how fast a particular elimination algorithm converges to the best (or close to the best) ordering. Our heuristic scoring function can perhaps be improved by adding more partial scores and combining them with weights estimated by regression techniques from performance data observed on several descriptors. Finally, DNA sequences are non-uniform, and a scheme that could adapt to changing character of sequences as they are processed would likely lead to further improvement of our algorithm. An interesting application of our algorithm would be assigning basic structural motifs to sequences as they are produced by high-throughput sequencers.

A logical extension of our problem is to construct descriptors automatically from known examples of RNAs. A step in this direction has already been taken and several algorithms to locate common substructures of two RNAs were developed [46, 47]. Such patterns are then basis of several practical tools for pattern-based RNA comparison, including ExpaRNA [48], LocARNA [49], and ExpaRNA-P [50].

## Ethics approval and consent to participate

not applicable.

## Consent for publication

not applicable.

## Availability of data and material

Software is available at http://compbio.fmph.uniba.sk/rnarobo. Data sets are publicly available as outlined in the paper. Descriptors for the searches are provided in Additional file 1.

## Additional file

Additional file 1 Supplementary online material. The file contains supplementary material with additional details on methods, file formats, and experiments. (PDF 617 kb)
