# Supplementary material for: RNA motif search with data-driven element ordering
Source: BMC Bioinformatics. 2016 May 18;17:216. doi: 10.1186/s12859-016-1074-x (PMC4870747; doi:10.1186/s12859-016-1074-x)
Supplement: Supplementary file 1 — Supplementary online material. The file contains supplementary material with additional details on methods, file formats, and experiments. (PDF 617 kb) [file 12859_2016_1074_MOESM1_ESM.pdf]

# RNA Motif Search With Data-Driven Element Ordering
# Supplementary Online Material

Ladislav Rampášek[1,2,3], Randi M. Jimenez[2], Andrej Lupták[2],
Tomáš Vinař[3], and Broňa Brejová[3]

[1] Department of Computer Science, University of Toronto, Toronto, ON M5R 3G4 Canada,
e-mail: rampasek@cs.toronto.edu
[2] Department of Pharmaceutical Sciences, Chemistry and Molecular Biology, University of California, Irvine, 2141 Natural Sciences 2, Irvine, CA 92697, e-mail: randij@uci.edu, aluptak@uci.edu
[3] Faculty of Mathematics, Physics, and Informatics, Comenius University, Mlynská dolina, 842 48 Bratislava, Slovakia e-mail: vinar@fmph.uniba.sk, brejova@dcs.fmph.uniba.sk

## Contents

**S1 RNArobo Descriptor Format** **1**

**S2 Dynamic Programming Recurrences** **3**

**S3 Information content heuristic** **4**

**S4 DDEO Performance** **7**

**S5 Extended Description of Experiments** **7**
- S5.1 GTP aptamer class I . . . 8
- S5.2 HHR type I in *Yarrowia lipolytica*. . . . 8
- S5.3 HHR type II in *Bacillus cereus* genome . . . 12
- S5.4 HDV-like ribozyme in *Anopheles gambiae* chr2L sequence . . . 12
- S5.5 HDV-like ribozymes in *Strongylocentrotus purpuratus* genome . . . 12

**S6 Descriptors for running time comparison** **18**
- S6.1 RNArobo descriptors . . . 18
- S6.2 RNAMot descriptors . . . 19
- S6.3 RNAmotif descriptors . . . 24

**S7 Comparison with RaligNAtor** **29**

## S1 RNArobo Descriptor Format

The format of an RNArobo descriptor is an extension of the descriptor format used by RNAbob (Eddy, 1996); thus RNAbob descriptors are compatible with RNArobo. A descriptor consists of three parts:

1. a **motif map** – a list of individual *structural elements* ordered from 5' to 3' end along the sequence
2. a detailed **specification** of each structural element

3. an optional *search order*

Each structural element is either single stranded (denoted by `s`) or helical (denoted by `h` or `r`). Detailed specification of each element consists of the following parts (the fields in bold are **mandatory**, while the fields in italic are *optional*):

(1.) the number of **mismatches** allowed (in helical elements, mismatches are allowed only on the positive strand),

(1b.) the number of **mispairs** allowed (for helical elements only),

(2.) the number of single nucleotide *insertions* allowed,

(3.) **primary sequence** constraints: a string composed of IUPAC nucleotide codes and wild cards "`*`". A wild card matches one nucleotide or none. Alternatively, an abbreviation for e.g. 10 wild cards can be written as "`[10]`",

(3b.) primary **sequence constraints** for the **negative strand** of a helical element. In helical elements, wild cards can occur only in pairs, i.e. for every wild card there must be a corresponding wild card on the other strand at the exactly opposite position,

(4.) IUPAC nucleotide code for *allowed insertions*,

(5.) a **transformation** string specifying pairings allowed in a *relational* element of type `r` (see details below).

Formatting of these fields is illustrated on the following simple motif composed of two elements: a helix `h1` capped by a single strand `s1`.

```
motif map
h1 s1 h1'

     # mismatches   # mispairs   positive strand   negative strand
h1       1       :      0          NNN**CC      :    GG**NNN

     # mismatches   sequence constraint
s1       0             ACCRNNT
```

Unlike RNAbob, RNArobo allows nucleotide insertions in individual elements. Syntax for specifying insertions is similar to the specification of the maximum number of mismatches or mispairs. The user has to specify the maximum number of insertions in the structural element and the identity of inserted nucleotides in the IUPAC code. Insertions are not allowed to occur at the very beginning and end of the matched regions, and insertions in helical regions cannot be adjacent nor opposite.

The following example demonstrates the use of insertions in a descriptor:

```
h1 s1 h1'
h1 0:0:2  NNN**CC:GG**NNN:A
s1 0:1    ACCRNNT:Y
```

In the `h1` helix we allow up to 2 insertions of adenosine, while in the single stranded element `s1` only one insertion of a pyrimidine nucleotide is allowed ('`Y`' stands for cytosine or thymine/uracil).

To specify a custom pairing function for a helical element, a *relational* element of type `r` can be used instead of a standard helix of type `h`, as in this variant of the previous descriptor:

```
r1 s1 r1'
r1 0:0:2  NNN**CC:GG**NNN:A  TGCA
s1 0:1    ACCRNNT:Y
```

The relational element `r1` allows only canonical base-pairs `A-T` and `C-G`. The individual IUPAC codes in the *transformation* string `TGCA` define nucleotides that can pair with `A`, `C`, `G`, and `T`, respectively, in this order. For default helical elements of type `h`, RNArobo allows also `G-U` wobble pair, as the default transformation string is `TGYR`.

The last line of a descriptor can contain an optional reorder command, which specifies the order in which elements are internally searched by the RNArobo algorithm, similarly to RNAMot Gautheret *et al.* (1990). If this command is absent or does not contain all elements, the automatic data-driven method is used to determine the best possible ordering of all remaining elements. This command has no principal impact on the actual results of the search, but defining a previously trained order can speed up the search by few seconds. Here is the previous descriptor with the element ordering line added:

```
r1 s1 r1'
r1 0:0:2  NNN**CC:GG**NNN:A  TGCA
s1 0:1    ACCRNNT:Y
R s1 r1
```

# S2 Dynamic Programming Recurrences

In this section, we describe the dynamic programming recurrences for finding all matches of a single element from the descriptor in a sequence window. We start by describing the algorithm for single-strand elements. Let us consider finding matches of a single-stranded pattern $P$ in a text $T$ with at most $M$ mismatches and $I$ insertions of a single-letter pattern $P_I$.

We use four dimensions of a five-dimensional table $S$ to keep track of position in $T$, position in $P$, the number of occurred mismatches, and the number of insertions, respectively. The fifth dimension is binary, and is intended to serve as a flag, whether the previous aligned symbol of $T$ is an insertion, as one insertion cannot follow another.

Formally, we define a binary function $S_{t,p,m,i,b}$ as follows:

$$t \in \{0 \dots |T|\}, p \in \{0 \dots |P|\}, m \in \{0 \dots M\}, i \in \{0 \dots I\}, b \in \{0, 1\}$$

$$S_{t,p,m,i,b} = \begin{cases} 1 & \text{if } P[1 \dots p] \text{ can be aligned with a suffix of } T[1 \dots t] \\ & \quad \text{with } m \text{ mismatches, and } i \text{ insertions;} \\ & \quad \text{if } b = 1,\ T[t] \text{ is an insertion} \\ & \\ 0 & \text{otherwise} \end{cases}$$

We start by computing values of $S$ for the empty prefix of the pattern, using the initial condition

$$\forall t \in \{0 \dots |T|\} \;\; S_{t,0,0,0,1} = 1.$$

The remaining values are computed using the following recurrence:

$$S_{t,p,m,i,0} = \bigvee \begin{cases} \bigvee_b S_{t-1,p-1,m-x,i,b} & \text{where } x = [T[t] \text{ does not fit } P[p]] \\ & \\ S_{t,p-1,m,i,0} & \text{if } P[p] = `*\text{' (skip a wild card)} \end{cases}$$

$$S_{t,p,m,i,1} = \bigvee \begin{cases} S_{t-1,p,m,i-1,0} & \text{if } T[t] \text{ fits } P_I \text{ (an insertion)} \\ & \\ S_{t,p-1,m,i,1} & \text{if } P[p] = `*\text{' (skip a wildcard)} \end{cases}$$

A match of the pattern $P$ is found in the text $T$ ending at position $t \le |T|$ with $m \le M$ mismatches and $i \le I$ insertions if $S_{t,|P|,m,i,0} = 1$.

Now we turn our attention to the more complex case of paired elements. The problem is to find all occurrences of a paired pattern $P : P'$ where $P$, $P'$ are patterns for individual strands (where $|P| = |P'|$) in a text $T$. Furthermore, we allow for imperfect matches with up to $M$ mismatches, $R$ mispairings, and with at most $I$ insertions of a single-letter pattern $P_I$ together in both strands.

To address this pattern matching problem, we introduce a function $H$, and a recurrence formula for its computation. The binary function $H_{t_1,t_2,p,m,r,i,b}$ for paired (helical) elements is the following:

$$t_1, t_2 \in \{0 \ldots |T|\}, p \in \{0 \ldots |P|\}, m \in \{0 \ldots M\}, r \in \{0 \ldots R\}, i \in \{0 \ldots I\}, b \in \{0, 1\}$$

$$H_{t_1,t_2,p,m,r,i,b} = \begin{cases} 1 & \text{if } P[1 \ldots p] \text{ can be aligned with a suffix } T' \text{ of } T[1 \ldots t_1] \text{ with} \\ & m \text{ mismatches, } P'[1 \ldots p] \text{ can be aligned with a prefix } T'' \\ & \text{of } T[t_2 \ldots |T|] \text{ with no mismatch, } T' \text{ and } T'' \text{ contain together} \\ & i \text{ insertions, and between } T' \text{ and } T'' \text{ are } r \text{ mispairings;} \\ & \text{if } b = 1 \text{, exactly one of } T[t_1], T[t_2] \text{ is an insertion} \\ & \qquad \textit{else} \text{ none of the } T[t_1] \text{ and } T[t_2] \text{ is an insertion} \\ & \\ 0 & \text{otherwise} \end{cases}$$

Again, we start with initial conditions for the empty prefix of the pattern:

$$\forall t_1, t_2 \in \{0 \ldots |T|\} \;\; H_{t_1,t_2,0,0,0,0,1} = 1$$

The recurrence for the remaining values works as follows:

$$\begin{aligned} \text{let} \quad & x = [T[t_1] \text{ does not fit } P[p]] \\ & y = [T[t_2] \text{ is not complement of } T[t_1]] \end{aligned}$$

$$H_{t_1,t_2,p,m,r,i,0} = \bigvee \begin{cases} \bigvee_b H_{t_1-1,t_2+1,p-1,m-x,r-y,i,b} & \text{if } T[t_2] \text{ fits } P'[p] \\ & \\ H_{t_1,t_2,p-1,m,r,i,0} & \text{if } P[p] = \text{`}*\text{'}^1 \text{ (skip a wildcard)} \end{cases}$$

$$H_{t_1,t_2,p,m,r,i,1} = \bigvee \begin{cases} H_{t_1-1,t_2,p,m,r,i-1,0} & \text{if } T[t_1] \text{ fits } P_I \text{ (an insertion)} \\ & \\ H_{t_1,t_2+1,p,m,r,i-1,0} & \text{if } T[t_2] \text{ fits } P_I \text{ (an insertion)} \\ & \\ H_{t_1,t_2,p-1,m,r,i,1} & \text{if } P[p] = \text{`}*\text{'}^1 \text{ (skip a wildcard)} \end{cases}$$

A match of the pattern $P : P'$ is found in the text $T$, $P$ ending at position $t_1 \leq |T|$, $P'$ beginning at position $t_2 \leq |T|$ with $m \leq M$ mismatches, $r \leq R$ mispairs, and $i \leq I$ insertions if $H_{t_1,t_2,|P|,m,r,i,0} = 1$.

# S3 Information content heuristic

In this section, we describe the details of the information content heuristic function $h_1$ omitted from the main text (Section "Element Ordering"). This function is an approximation of the information content of an element, favoring elements that pose more specific constraints.

For a single-stranded element $S$, let $N$ be the length of its longest possible match and let $X$ be the number of sequences of length $N$ that contain a match of the element starting at the first position. As explained in the main text, the information content of element $S$ is then estimated as $h_1(S) = 2N - \log_2 X$.

[1] In a correct paired element, we have $P[k] =$ '$*$' if and only if $P'[k] =$ '$*$'.

Since the value of $X$ is hard to compute for complex elements, we instead use an upper bound $X_U \geq X$. To obtain the upper bound, we count different ways of obtaining a sequence matching $S$, disregarding the fact that some sequences may be obtained in several different ways and consequently counted multiple times.

In the simplest case, element $S$ does not contain any flexible-length wild cards and does not allow for any distortions (mismatches, insertions). The element specifies for each position $i$ the set of allowed nucleotides; let $C[i]$ be the size of this set. The value of $X$ is then simply

$$X = \prod_{i=1}^{N} C[i]. \tag{1}$$

Next we extend the bound to cases when $S$ contains wild cards. Each wild card corresponds to an arbitrary nucleotide or to an empty string. A block of $k$ consecutive wild cards thus corresponds to an arbitrary sequence of length up to $k$. Let $X_1$ be the value obtained by formula (1) if we consider only non-wild card positions in $S$. A single block of $k$ consecutive wild cards increases the value of $N$ (the length of the longest occurrence of $S$) by $k$. These $k$ additional nucleotides can be arbitrary, and are split into a block of length $i$ matching the block of wild cards and a block of length $k-i$ located after the element occurrence (this block corresponds to the unused wild cards). Since the value of $i$ can be any integer between 0 and $k$, this leads to the upper bound of $X_1(k+1)4^k$ sequences matching $S$. If $S$ has multiple blocks of wild cards of lengths $k_1, \ldots, k_b$, each of them can be split into two blocks independently, leading to the upper bound

$$X_2 = X_1 \cdot \prod_{i=1}^{b} 4^{k_i}(k_i + 1). \tag{2}$$

Next, let us assume that element $S$ allows up to $M_M$ mismatches. Let $N'$ be the number of positions where a mismatch can occur (in this count we omit wild cards as well as positions where $S$ allows any nucleotide). Let us denote by $A$ the set of positions where mismatches actually occur (the size of $A$ is at most $M_M$). There are $\prod_{i \in A} C[i]$ ways to place matching nucleotides at positions in $A$ and $\prod_{i \in A}(4 - C[i])$ ways to place mismatches at those positions. We could obtain our estimate by enumerating all sets $A$ of size at most $M_M$:

$$X_3' = X_2 \sum_{A: 0 \leq |A| \leq M_M} \prod_{i \in A} \left( \frac{4 - C[i]}{C[i]} \right). \tag{3}$$

Instead, we could use an upper bound by assuming $C[i]$ to be 1 at every position, allowing for a simpler formula.

$$X_3' = X_2 \sum_{j=0}^{M_M} \binom{N'}{j} 3^j. \tag{4}$$

In the real application, we instead replace $C[i]$ by the empirical mean $\bar{C}$ of all the positions where a mismatch can occur, obtaining an expression which is not guaranteed to be an upper bound of $X$, but works well in practice:

$$\bar{C} = \frac{1}{N'} \sum_{i=1}^{N'} C[i], \tag{5}$$

$$X_3 = X_2 \sum_{j=0}^{M_M} \binom{N'}{j} \left( \frac{4 - \bar{C}}{\bar{C}} \right)^j. \tag{6}$$

Finally, we address the case where $S$ allows up to $M_I$ insertions. The nucleotides to be inserted are constrained in the descriptor to come from some set of size $C_{ins}$. Let $N''$ be the number of positions in $S$,

where an insertion can occur. If the actual number of insertions is $j$, they can be inserted in $\binom{N''}{j} C_{ins}^{j}$ ways. Similarly to unused wild cards, we have to add padding for each unused insertion to achieve sequences of total length $N$. Thus we obtain the following approximate upper bound $X_U$ for count $X$:

$$X_U = X_3 \sum_{j=0}^{M_I} \binom{N''}{j} C_{ins}^{j} 4^{M_I - j}. \tag{7}$$

Computation for paired element is analogous. Let $H$ be an element consisting of two paired strands $H_1$ and $H_2$, and let $N$ be the maximum length of a match to one of these two strands, after accounting for wild cards and insertions. Let $X$ be the number of pairs of sequences of length $N$ such that $H_1$ occurs in the first sequence starting at the first position, and $H_2$ occurs in the second sequence ending at the last position, and these two occurrences satisfy the complementarity constraints with up to allowed amount of distortion. We again compute an approximate upper bound $X_U$ instead of the number $X$, counting different ways that such a matching can occur. Then we use the following estimate of the information content of a paired element $H$:

$$h_1(H) = 4N - \log_2 X_U. \tag{8}$$

As with single-stranded elements, we first count the number of sequences that match $H$ without considering wild cards and distortions. Let $P[i]$ be the number of valid base pairs between position $i$ of $H_1$ and the corresponding position of $H_2$. The value of $P[i]$ is determined by both complementarity constraints specified by $H$ and by sequence constraints for the respective positions in $H_1$ and $H_2$. As before, the total number of matching sequences is the product

$$X_1 = \prod_{i=1}^{N} P[i]. \tag{9}$$

Wild cards occur in symmetrical positions of $H_1$ and $H_2$, and each block of $k$ wild cards has to be matched by $j \le k$ valid base pairs. Therefore, the estimate for $b$ blocks of wild cards with lengths $k_1, \dots, k_b$ is

$$X_2 = X_1 \cdot \prod_{i=1}^{b} \sum_{j=0}^{k_i} P^j \cdot 16^{k_i - j}. \tag{10}$$

In this expression, $P$ is the number of admissible base pairs, for example 4, if we require strict Watson-Crick pairs, or 6, if we allow U-G pairs as well. The factor $16^{k_i-j}$ comes from the need to pad each of the two sequences with $k_i - j$ nucleotides.

Mismatches are allowed only on the positive strand $H_1$ of $H$. Let $P'[i]$ be the same as $P[i]$ except that we disregard sequence constraints specified for position $i$ in $H_1$. Therefore $P'[i] - P[i]$ is the number of base pairs that form a potential mismatch at position $i$ (satisfying the complementarity constraints). As before, we could enumerate all possible sets $A$ of mismatch positions of size up to $M_M$:

$$X_3' = X_2 \sum_{A:0 \le |A| \le M_M} \prod_{i \in A} \left( \frac{P'[i] - P[i]}{P[i]} \right). \tag{11}$$

To simplify the formula, we could again use an upper bound on the term $(P'[i] - P[i])/P[i]$, but in practice we approximate the actual value of $P[i]$ by the empirical mean $\bar{P}$ and we approximate $P'[i]$ by the total number of base pairs $P$. Thus we obtain the following estimate:

$$\bar{P} = \frac{1}{N'} \sum_{i=1}^{N'} P[i], \tag{12}$$

$$X_3 = X_2 \sum_{j=0}^{M_M} \binom{N'}{j} \left( \frac{P - \bar{P}}{\bar{P}} \right)^j. \tag{13}$$

As before, $N'$ is the number of positions in $H_1$ where a mismatch can occur.

Paired elements may also allow up to $M_P$ mispairs, where the two paired nucleotides do not form a valid base pair. Mispairs are treated similarly as mismatches; value $P'[i]$ is replaced by 16, which is the number of all possible pairs. Value $P[i]$ is again approximated by its mean $\bar{P}$. Unlike mismatches, mispairs can occur at all positions of the motif, including portions matching wild cards. The number of such positions is $N'' = N - M_I$, where $M_I$ is the number of allowed insertions. We obtain the following bound

$$X_4 = X_3 \sum_{j=0}^{M_P} \binom{N''}{j} \left( \frac{16 - \bar{P}}{\bar{P}} \right)^j . \tag{14}$$

Finally, if element $H$ allows up to $M_I$ single nucleotide insertions, sequence of each strand has space for $M_I$ insertions, and therefore any of these $2M_I$ positions not used as insertions need to be padded by arbitrary bases. For simplicity, we ignore the restriction that positions of two insertions should not be opposite in a helix. This gives us the final value $X_U$ used in computing the information content heuristic.

$$X_U = X_4 \sum_{j=0}^{M_I} \binom{2N''}{j} C_{ins}^{j} 4^{(2M_I - j)}. \tag{15}$$

# S4 DDEO Performance

Here, we demonstrate the performance of the DDEO heuristic on the hepatitis delta virus like ribozyme (HDV) motif (Fig.4), which is the most complex motif that we used in our experiments. The motif contains four helical paired elements and six single stranded elements, organized in a double pseudoknot.

Figures S1 and S2 show details of DDEO operation with the HDV ribozyme descriptor over five runs of RNArobo on the human genome. Both figures show data for all element orderings selected for the initial candidate set $O$. Fig.S1 shows that the heuristic score is not perfect; although many good orderings were included in the set, they are mixed with much worse orderings. Fig.S2 shows the number of samples necessary to eliminate a particular ordering. For orderings with a bad performance, we typically need very few samples (as few as two in some cases), while the orders with performance close to the optimum are tried many times, but since their performance is good, their repeated use does not increase the overhead significantly.

# S5 Extended Description of Experiments

RNArobo uses the descriptor format of RNAbob (Eddy, 1996), but extends it with the option of allowing insertions, which is particularly useful for representing bulges in helical elements. Here we demonstrate the utility of our tool on several data sets containing functional ribozyme occurrences reported in literature (Webb *et al.*, 2009; Webb and Lupták, 2011; Perreault *et al.*, 2011). For every RNA motif, the same descriptor was used for search with both RNArobo and RNAbob. RNArobo was also run with a variant of each descriptor allowing insertions in helical regions. In several cases, these modified descriptors allowed us to discover additional known or putative ribozyme occurrences. The results are summarized in Table 1 and described in detail below.

The results of RNA motif searches often contain false positives, whose sequence satisfies the restrictions given in the descriptor but would not form the desired secondary structure if transcribed. To filter out such false positives, we have used our post-processing pipeline named Fold-Filter (Jimenez *et al.*, 2012), which is provided as a part of the RNArobo 2.1 package. This pipeline predicts secondary structure and its stability using ViennaRNA package (Lorenz *et al.*, 2011) and DotKnot (Sperschneider and Datta, 2010) and compares the results with user-defined thresholds. The last three experiments illustrate the use of this pipeline for improving specificity of the search.

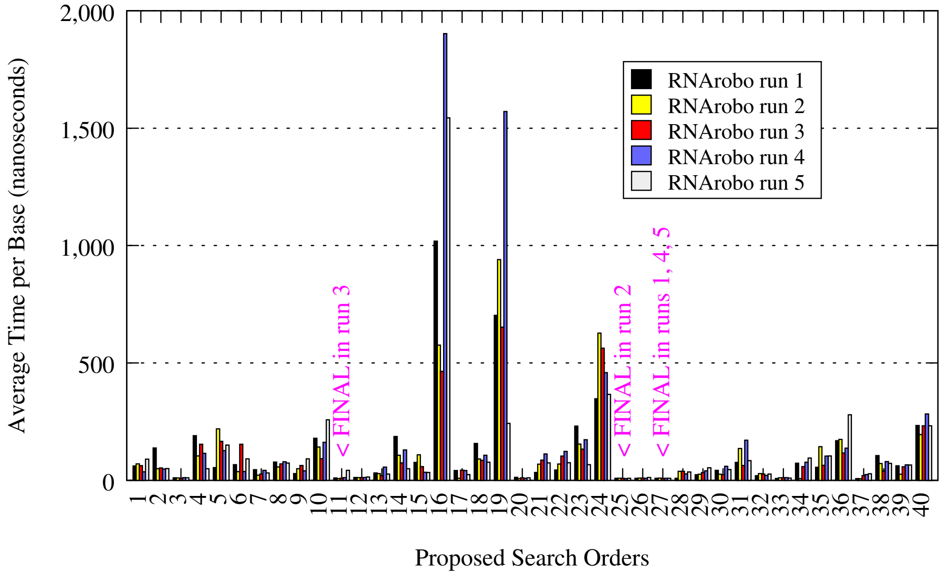


Figure S1: The average time $T_x$ used by the first 40 triplets with the best heuristic score (ordered by the heuristic score from left to right) in five runs of RNArobo search in the human genome for the HDV ribozyme descriptor. The final element orderings selected in each run are highlighed.

## S5.1 GTP aptamer class I

In this experiment, we search a compilation of sequences acquired through in vitro selections for GTP aptamers by Szostak and coworkers (Davis and Szostak, 2002). We have constructed a descriptor for the GTP aptamer class I encompassing both the conserved sequence and secondary structure facilitating ligand-aptamer binding (Carothers *et al.*, 2006). The descriptor for the search permitting insertions limits them to adenosine only. Both RNAbob and RNArobo discover nine unique sequences fitting the descriptor, but after allowing insertions, RNArobo finds one new instance. Since the library was selected for GTP binding, we assume that all these instances are functional.

## S5.2 HHR type I in *Yarrowia lipolytica.*

The hammerhead ribozyme (HHR) motif is characterized by three helices anchored in a sequence conserved-catalytic core (Perreault *et al.*, 2011). These structure descriptors (hhr1-4bp and hhr1-4bp-ins) were used to search through the yeast *Yarrowia lipolytica* CLIB122 (NC_006067.1-NC_006072.1) genome. This genome contains several type I HHRs (Perreault *et al.*, 2011). Both RNAbob and RNArobo found a single HHR (Yli-1-3 position 1037945-1037850) using a descriptor with a strict requirement for at least four base pairs in each helix (hhr1-4bp). An RNArobo search using the same descriptor, but allowing single-nucleotide insertions in each of the three helices (hhr1-4bp-ins), increased the number of unique hits from one to fifteen. Among these hits were Yli-1-3, and also Yli-1-4 through Yli-1-11 ribozymes. The search allowing insertions was necessary for finding the eight additional ribozymes.

Decreasing the requirement in base pairing of helices from four to three base-pairs resulted in a total of four hits from both RNAbob and RNArobo searches (hhr1-3bp), including Yli-1-3 and Yli1-13, which was not found when helices were required to be at least four base pairs long. Allowing for single nucleotide insertions in any of the three helices of the HHR (hhr1-3bp-ins) significantly increased the number of hits returned by RNArobo from four to fifty-four, including the same known ribozymes (Yli-1-3 through Yli-1-11 and Yli-1-13). A sequence alignment of the output reveals that many sequences can be grouped into two main families. The first family includes the known "Yli" ribozymes, and the second includes previously unidentified putative ribozymes. Further analysis of this second family was done using the Fold-Filter script included with the RNArobo-2.0 package.

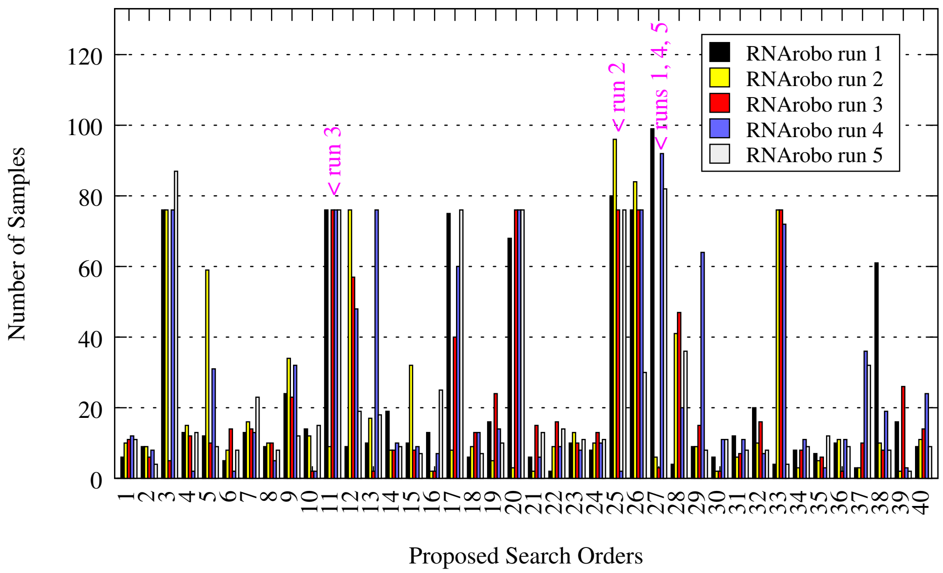


Figure S2: The number of samples for each of the 40 triples from Fig.S1 before it was eliminated or chosen as the best element ordering.

```
h1  s1  h2  s2  h2'  s3  h1'
h1  0:0  **NNNN:NNNN**
s1  2    *AAGTGGTTGGG*
h2  0:0  **NNN:NNN**
s2  1    *UUCG*
s3  2    *UGUGAAAA*
```

Figure S3: Descriptor for GTP aptamer class 1

Each of the ten sequences in the second family, although not necessarily unique in sequence, is unique in the genomic location, making these ten putative ribozymes independent findings (Figures S7 and S8). In this instance, RNArobo may have found a previously unidentified family of type I HHRs in the *Y. lipolytica* genome. This is significant in that no other sequence-conserved family of HHR is known to exist in this genome, and the new family is similar to the large family of HHRs in the *Schistosoma mansoni* genome.

```
h1  s1  h2  s2  h2'  s3  h1'
h1  0:0:1  **NNNN:NNNN**:A
s1  2      *AAGTGGTTGGG*
h2  0:0:1  **NNN:NNN**:A
s2  1      *UUCG*
s3  2      *UGUGAAAA*
```

Figure S4: Descriptor for GTP aptamer class 1 with insertions

```
s1 h1 s2 h2 s3 h2' s4 h3 s5 h3' s6 h1' s7
h1 0:0 NNNN*:*NNNN
h2 0:0 NNNN*:*NNNN
h3 0:0 NNNN*:*NNNN
s1 0   NNNNNNNNNNNNNNN
s2 0   CTGANGA
s3 0   NNNN[10]
s4 0   GAAA
s5 0   NNNN[10]
s6 0   NH
s7 0   NNNNNNNNNNNNNNN
```

Figure S5: Descriptor for HHR type I

```
s1 h1 s2 h2 s3 h2' s4 h3 s5 h3' s6 h1' s7
h1 0:0:1 NNNN*:*NNNN
h2 0:0:1 NNNN*:*NNNN
h3 0:0:1 NNNN*:*NNNN
s1 0     NNNNNNNNNNNNNNN
s2 0     CTGANGA
s3 0     NNNN[10]
s4 0     GAAA
s5 0     NNNN[10]
s6 0     NH
s7 0     NNNNNNNNNNNNNNN
```

Figure S6: Descriptor for HHR type I with insertions

```
> 43 2639305 2639226 gi|50557461|ref|NC_006069.1|  Yarrowia lipolytica CLIB122 chromosome C
ATTAACTTAGAACAGGACCCTGAAGACTGCTACCAAGTGGGAAATGGTTGGCACTACCTGACGTCGGACGCCCTCACTGA
> 44 2699756 2699677 gi|50557461|ref|NC_006069.1|  Yarrowia lipolytica CLIB122 chromosome C
ATTAACTTAGAACAGGACCCTGAAGACTGCTACCAAGTGGGAAATGGTTGGCACTACCTGACGTCGGACGCCCTCACTGA
> 1 457449 457370 gi|50557458|ref|NC_006072.1|  Yarrowia lipolytica CLIB122 chromosome F
ATTAACTTAGAACAGGACCCTGAAGACTGCTACCAAGTGGGAAATGGTTGGCACTACCTGACGTCGGACGCCCTCACTGA
> 29 3516159 3516238 gi|50557462|ref|NC_006070.1|  Yarrowia lipolytica CLIB122 chromosome D
ATTAACTTAGAACAGGACCCTGAAGACTGCTACCAAGTGGGAAATGGTTGGCACTACCTGACGTCGGACGCCCTCACTGA
> 31 458931 459010 gi|50557461|ref|NC_006069.1|  Yarrowia lipolytica CLIB122 chromosome C
ATTAACTTAGAACAGGACCCTGAAGACTGCTACCAAGTGGGAAATGGTTGGCACTACCTGACGTCGGACGCCCTCACTGA
> 35 1506845 1506766 gi|50557461|ref|NC_006069.1|  Yarrowia lipolytica CLIB122 chromosome C
ATTAACTTAGAACAGGACCCTGAAGACTGCTACCAAGTGGGAAATGGTTGGCACTACCTGACGTCGGACGCCCTCACTGA
> 17 287901 287980 gi|50557462|ref|NC_006070.1|  Yarrowia lipolytica CLIB122 chromosome D
ACTAACTTAGAACAGGACCCTGAAGACTGCTACCAAGTGGGAAATGGTTGGCACTACCTGACGTCGGACGCCCTCACTGA
> 54 1084138 1084217 gi|50557456|ref|NC_006067.1|  Yarrowia lipolytica CLIB122 chromosome A
ACTAACTTAGAACAGGACCCTGAAGACTGCTACCAAGTGGGAAATGGTTGGCACTACCTGACGTCGGACGCCCTCACTGA
> 20 759429 759508 gi|50557462|ref|NC_006070.1|  Yarrowia lipolytica CLIB122 chromosome D
ACTAACTTAGAACAGGACCCTGAAGACTGCTACCAAGTGGGAAATGGTTGGCACTACCTGACGTCGGACGCCCTCACCGA
> 51 708177 708098 gi|50557456|ref|NC_006067.1|  Yarrowia lipolytica CLIB122 chromosome A
ACTAACTTAGAACAGGACCCTGAAGACTGCTACCAAGTGGGAAATGGTTGGCACTACCTGACGTCGGACGCCCTCACCGA
```

Figure S7: A new previously unidentified putative family of type I HHRs in the *Y. lipolytica* genome. Each of the ten sequences in the family, although not necessarily unique in sequence, is unique in the genomic location, making these ten putative ribozymes independent findings.

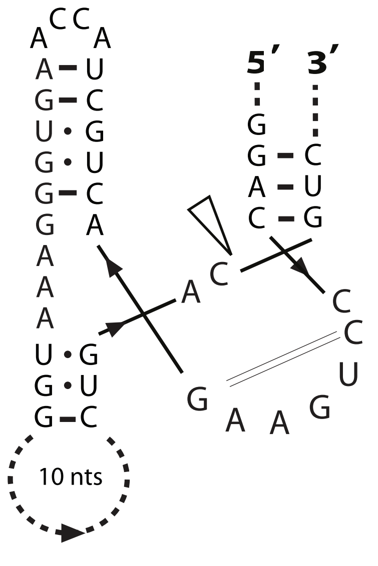


Figure S8: Putative secondary structure of the novel HHRs from *Y. lipolytica*.

### S5.3 HHR type II in *Bacillus cereus* genome

Type II HHRs differ from type I only by the stem containing the 5' and 3' termini of the RNA. These descriptors (hhr2-3bp and hhr2-3bp-ins) were used to search for type II HHRs in the bacterium *Bacillus cereus* ATCC 14579 genome (NC_004722.1) (Ivanova *et al.*, 2003), which has one known example of this ribozyme. Both RNAbob and RNArobo yielded a single sequence corresponding to the known HHR Bce-1-1. The RNArobo search allowing insertions (hhr2-3bp-ins) increased the number of hits from one to four. The Fold-Filter script, based on the Vienna RNAfold algorithm did not predict optimal secondary structure formation for any of the three additional hits, suggesting that these may not be bona fide ribozymes.

### S5.4 HDV-like ribozyme in *Anopheles gambiae* chr2L sequence

HDV-like ribozymes are characterized by a nested double-pseudoknot secondary structure with only six conserved nucleotides. The descriptors hdv-looseP4 and hdv-looseP4-ins were used to search chromosome 2L (NT_078265.2) of the mosquito *Anopheles gambiae* (Holt *et al.*, 2002). The genome of this mosquito is known to harbor multiple families of HDV-like ribozymes, many of which map to retrotransposable elements (Webb *et al.*, 2009; Ruminski *et al.*, 2011). These ribozymes were first discovered by an RNAbob search followed by sequence similarity search, which found additional examples not fitting the descriptor (Webb *et al.*, 2009).

Both RNAbob and RNArobo found known drz-Agam-1-1 ribozyme and an additional putative ribozyme which displays characteristic HDV-like secondary structure. No other output from these searches appeared to have features characteristic of canonical HDV-like ribozymes. The output from RNArobo allowing for insertions produced one additional known ribozyme, drz-Agam-1-2, which was previously identified through sequence similarity to Agam-1-1.

### S5.5 HDV-like ribozymes in *Strongylocentrotus purpuratus* genome

The genome of the purple sea urchin (Sodergren *et al.*, 2006) is known to contain many HDV-like ribozymes, which can be grouped into at least four sequence families (Webb *et al.*, 2009). Two different types of searches were conducted in this genome. The first was a loose search allowing region P4 to be variable (element `s5` in hdv-looseP4), resulting in numerous hits, many of which were further determined by Fold-Filter to be false positives. Of the fifteen confirmed ribozymes found, most group into two sequence families with a single outlier possibly belonging to a separate family. The RNArobo search allowing insertions (hdv-looseP4-ins) found one additional positive hit, which aligns with one of the two families seen previously (see sequence alignments in Figure S15 and S16 and structure in Figure S17).

A second, more restrictive search in the *S. purpuratus* genome included the P4 region in the defined secondary structure (element `h4` and `s5` in hdv-stemP4), the helical region being an essential feature of the

```
s1 h1 s2 h2 s3 h2' s4 h3 s5 h3' s6 h1' s7
h1  0:0  ******NNN:NNN******
h2  0:0  ******NNN:NNN******
h3  0:0  ******NNN:NNN******
s1  0    NNNNNNNNNNNNNNN
s2  0    GAAA
s3  0    NNNN[8]
s4  0    NH
s5  0    NNNN[8]
s6  0    CTGANGA
s7  0    NNNNNNNNNNNNNNN
```

Figure S9: Descriptor for HHR type II

```
s1 h1 s2 h2 s3 h2' s4 h3 s5 h3' s6 h1' s7
h1 0:0:1 ******NNN:NNN******:A
h2 0:0:1 ******NNN:NNN******:A
h3 0:0:1 ******NNN:NNN******:A
s1 0     NNNNNNNNNNNNNNN
s2 0     GAAA
s3 0     NNNN[8]
s4 0     NH
s5 0     NNNN[8]
s6 0     CTGANGA
s7 0     NNNNNNNNNNNNNNN
```

Figure S10: Descriptor for HHR type II with insertions

```
h1 s1 h3 r4 s2 r4' h1' s4 s5 s6 h3' s7
h1 0:0 RNNNN***:***NNNNY
s1 0   N[50]
h3 0:0 NNNNNN**:**NNNNNN
r4 0:0 NNN:NNN TGCA
s2 0   TYYHCG*Y
s4 0   RN
s5 0   NNNN[50]
s6 0   CNRA*
s7 0   NNNNNN
```

Figure S11: Descriptor for HDV-like ribozyme with loose P4 region

```
h1 s1 h3 r4 s2 r4' h1' s4 s5 s6 h3' s7
h1 0:0   RNNNN***:***NNNNY
s1 0     N[50]
h3 0:0:1 NNNNNN**:**NNNNNN
r4 0:0   NNN:NNN TGCA
s2 0     TYYHCG*Y
s4 0     RN
s5 0     NNNN[50]
s6 0     CNRA*
s7 0     NNNNNN
```

Figure S12: Descriptor for HDV-like ribozyme with loose P4 region and allowed insertions

```
h1 s1 h3 r4 s2 r4' h1' s4 h4 s5 h4' s7 h3' s8
h1 0:0 GNNNNN*:*NNNNNY
s1 0   N[50]
h3 0:0 NNNNNN**:**NNNNNN
r4 0:0 NNN:NNN TGCA
s2 0   TYYHCG*Y
s4 0   RN
h4 0:0 NNNNN*:*NNNNN
s5 0   NNN***
s7 0   CNRA*
s8 0   NNNNNN
```

Figure S13: Descriptor for HDV-like ribozyme with structured P4 region

```
h1 s1 h3 r4 s2 r4' h1' s4 h4 s5 h4' s7 h3' s8
h1 0:0   GNNNNN*:*NNNNNY
s1 0     N[50]
h3 0:0:1 NNNNNN**:**NNNNNN
r4 0:0   NNN:NNN TGCA
s2 0     TYYHCG*Y
s4 0     RN
h4 0:0:1 NNNNN*:*NNNNN
s5 0     NNN***
s7 0     CNRA*
s8 0     NNNNNN
```

Figure S14: Descriptor for HDV-like ribozyme with structured P4 region and allowed insertions

HDV ribozyme secondary structure with the length of the structure varying extensively. With the more restrictive search (hdv-stemP4 and hdv-stemP4-ins) all eleven hits are confirmed ribozymes.

```
26       GGGGGCCCCCGGGGGCCATTGAAG-GAGCGTTCACGTC--GCGGTCCC--TGTCA-GGT- 53
30       GGGGGCCCCCGGGGGCCATTGAAG-GAGCGTTCACGTC--GCGGTCCC--TGTCA-GAT- 53
25       -GGGGGCCCCGGGGGCCATTGAAG-GAGCGTTCACGTC--GCGGTCCC--TGTCA-GAT- 52
29       -GGGGGCCCCGGGGGCCATTGAAG-GAGCGTTCACGTC--GCGGTCCC--TGTCA-GAT- 52
6        -GGGGGTCCCGGGGGCCATTGAAG-GAGCGTTCACGTC--GCGGCCCC--TGTCA-GAT- 52
44       ----------GGGGGCCATTGAAG-GAGCGTTTACGTC--GCGGTCCC--TGCCA-GAT- 43
55       ----------GGGGGCCATTGAAG-GAGCGTTCACGTC--GCGGTCCC--TGTCA-GAT- 43
52       ----------GGGGGCCATTGAAG-GAGCGTTCACGTC--GCGGTCCC--TGTCA-GAT- 43
45       ----------GGGGGCCATTGAAG-GAGCGTTCACGTC--GCGGTCCC--TGTCA-GAT- 43
24       ----------GGGGGCCATTGAAG-GAGCGTTCACGTC--GCGGTCCC--TGTCA-GAT- 43
56       ----------GGGGGCCATTGAAG-GAGCGTTCACGTC--GCGGTCCC--TGTCA-GAT- 43
13       ----------GGGTTGCACAGGAG-CAGGGTCCACGTCCCGCAACCTGGGTGTCATGATT 49
8        -----------GGGGTTA-TGTTGTCGACCTTCACGTG--GTGACCC---TGTTA--ATT 41
39       -----------GGGGTTA-TGTTGTCGACCTTCACGTG--GTGACCC---TGTTA--ACT 41
46       -----------GGGGTTAATGTTGTCGACCTTCACGTG--GTGACCC---TGTTA--ACT 42
                    **    *  *  *      *  ****   *    *    **  *

26       ----GAAAATCTG----CGAATCCTTCAACGAA--AT-- 80
30       ----GAAAATCTG----CGAATCCTTCAACTAC--AC-- 80
25       ----GAAAATCTG----CGAATCCTTCAACTAC--AC-- 79
29       ----GAAAATCTG----CGAATCCTTCAACTAC--AC-- 79
6        ----GAAAATCTG----CGAATCCTTCAGCTAC--AC-- 79
44       ----GAAAATCTG----CGAATCCTTCAACCAC--AC-- 70
55       ----GAAAATCTG----CGAATCCTTCAACCAC--AC-- 70
52       ----GAAAATCTG----CGAATCCTTCAACCAC--AC-- 70
45       ----GAAAATCTG----CGAATCCTTCAACCAC--AC-- 70
24       ----GAAAATCTG----CGAATCCTTCAACCAC--AC-- 70
56       ----GAAAATCTG----CAAATCCTTCAACCAC--AC-- 70
13       TCTTGAAGCCATGATAGCTGATGCTCCTAC-AT--GT-- 83
8        -----GACTTCTG----TCAAACTAACGACAACCGATAA 71
39       -----GACTAATG----TCAAACTAACGACAACCGATAA 71
46       -----GACTACTG----TCAAACTAACGACAACCGATAA 72
               *    **       *     *  * *
```

Figure S15: ClustalW 2.1 multiple sequence alignment of HDV-like ribozyme structures found by both RNArobo and RNAbob in genome of *S. purpuratus*. The search was conducted with a descriptor that required strict helix in P1 region of the HDV ribozyme structure. Of the 15 confirmed ribozymes found, most group into two clear families (top 11 and bottom 3 sequences, respectively) with a single outlier possibly belonging to a separate family (12th sequence in our alignment).

```
511     GGGGGCCCCCGGGGGCCATTGAAGGAGCGTTCACGTC--GCGGTCCC--TGTCA-GGT-- 53
620     GGGGGCCCCCGGGGGCCATTGAAGGAGCGTTCACGTC--GCGGTCCC--TGTCA-GAT-- 53
510     -GGGGGCCCCGGGGGCCATTGAAGGAGCGTTCACGTC--GCGGTCCC--TGTCA-GAT-- 52
619     -GGGGGCCCCGGGGGCCATTGAAGGAGCGTTCACGTC--GCGGTCCC--TGTCA-GAT-- 52
135     -GGGGGTCCCGGGGGCCATTGAAGGAGCGTTCACGTC--GCGGCCCC--TGTCA-GAT-- 52
1023    ----------GGGGGCCATTGAAGGAGCGTTCACGTC--GCGGTCCC--TGTCA-GAT-- 43
820     ----------GGGGGCCATTGAAGGAGCGTTCACGTC--GCGGTCCC--TGTCA-GAT-- 43
794     ----------GGGGGCCATTGAAGGAGCGTTCACGTC--GCGGTCCC--TGTCA-GAT-- 43
509     ----------GGGGGCCATTGAAGGAGCGTTCACGTC--GCGGTCCC--TGTCA-GAT-- 43
775     ----------GGGGGCCATTGAAGGAGCGTTTACGTC--GCGGTCCC--TGCCA-GAT-- 43
1024    ----------GGGGGCCATTGAAGGAGCGTTCACGTC--GCGGTCCC--TGTCA-GAT-- 43
334     ----------GGGTTGCACAGGAGCAGGGTCCACGTCCCGCAACCTGGGTGTCATGATTT 50
733     ----------GGGGTTA-TGTTGTCGACCTTCACGTG--GTGA-CCC--TGTTA--ACT- 41
796     ----------GGGGTTAATGTTGTCGACCTTCACGTG--GTGA-CCC--TGTTA--ACT- 42
185     ----------GGGGTTA-TGTTGTCGACCTTCACGTG--GTGA-CCC--TGTTA--ATT- 41
723     ----------GGGGTTCATGTTGTCGACCTTCACGTG--GTGAGCCC--TGTCA--ACT- 43
                  ***                *  ****   *    *    **  *

511     ---GAAAATCTG----CGAATCCTT---CAACGAA-- 78
620     ---GAAAATCTG----CGAATCCTT---CAACTAC-- 78
510     ---GAAAATCTG----CGAATCCTT---CAACTAC-- 77
619     ---GAAAATCTG----CGAATCCTT---CAACTAC-- 77
135     ---GAAAATCTG----CGAATCCTT---CAGCTAC-- 77
1023    ---GAAAATCTG----CGAATCCTT---CAACCAC-- 68
820     ---GAAAATCTG----CGAATCCTT---CAACCAC-- 68
794     ---GAAAATCTG----CGAATCCTT---CAACCAC-- 68
509     ---GAAAATCTG----CGAATCCTT---CAACCAC-- 68
775     ---GAAAATCTG----CGAATCCTT---CAACCAC-- 68
1024    ---GAAAATCTG----CAAATCCTT---CAACCAC-- 68
334     CTTGAAGCCATGATAGCTGATGCTC---CTAC-ATG- 82
733     ----GACTAATG---TCAAACTAAC-GACAACCGATA 70
796     ----GACTACTG---TCAAACTAAC-GACAACCGATA 71
185     ----GACTTCTG---TCAAACTAAC-GACAACCGATA 70
723     ----GACTGCTG---TCAGGCTAACAGACAACCATTT 73
             *    **    *           *  *
```

Figure S16: ClustalW 2.1 multiple sequence alignment of HDV-like ribozyme structures found by RNArobo in genome of *S. purpuratus* when allowing an insertion in P1 helical region of the HDV ribozyme structure. This search found one additional positive hit, which aligns with the latter of the two families seen in the previous search (Fig.S15).

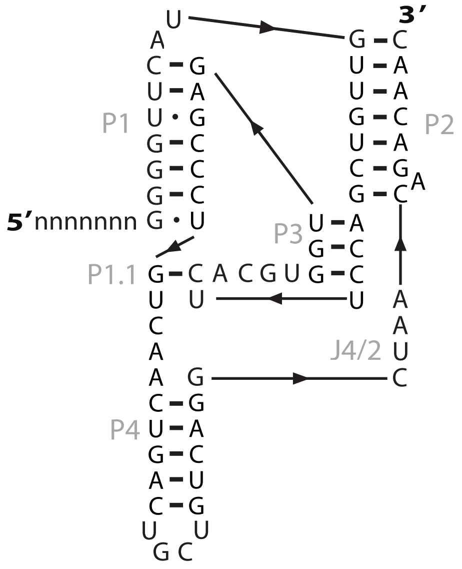


Figure S17: Putative secondary structure of the novel HDV from *S. purpuratus*.

## S6 Descriptors for running time comparison

Table 2 in the main text contains running time comparison of RNArobo with several other tools. RNAbob can be directly compared, as RNArobo uses an extended version of the same descriptor format. We have created descriptors for RNAmotif and RNAmot that match RNArobo descriptors as closely as possible, but there are some small differences. For example, RNAMot does allow mispairs in helical regions, and the number of allowed wobble pairs and base mismatches is parametrized globally in the whole motif, rather than per-element, as in RNArobo. In this section, we list all descriptors used in our experiments.

### S6.1 RNArobo descriptors

Most descriptors used in Table 2 for RNArobo and RNAbob are shown elsewhere in this supplement. In particular, GTP aptamer is in Figure S3, tRNA in Figure S42, HHR type I in Figure S5, HHR type II in Figure S9, HDV-like ribozyme with loose P4 region in Figure S11, and HDV-like ribozyme with structured P4 region in Figure S13. The remaining descriptors are shown below.

```
h1 r2 s1 r3 h4 s2 h4' r3' s3 r2' h1'

h1 0:0 *****:*****
r2 0:0 NNNN:NNNN TGCA
r3 0:0 CNNN:NNNG TGCA
h4 0:0 *****:*****

s1 0 GGAAGAAACTG
s2 0 NNN[17]
s3 0 G
```

Figure S18: Descriptor for ATP aptamer used with RNArobo and RNAbob.

```
s1 r1 s2 r2 s3 r2' s4 r3 s5 r3' s6 r1' s7

r1 0:0 ***NNN:NNN*** TGCA
r2 0:0 ***NNN:NNN*** TGCA
r3 0:0 ***NNN:NNN*** TGCA

s1 0   NNNNNNNNNN
s2 0   CTGANGA
s3 0   NNNN[46]
s4 0   GAAA
s5 0   NNNN[46]
s6 0   TN
s7 0   NNNNNNNNNN
```

Figure S19: Descriptor for HHR (extended) used with RNArobo and RNAbob.

```
h1 r2 s1 r3 s2 r4 r5 s3 r5' r2' h1' s4 h6 s5 h6' s6 r3' r4'

h1 0:0 R:Y
r2 0:1 NNNNNN:NNNNNN TGCA
r3 0:0 NNNN**:**NNNN TGCA
r4 0:0 NNN:NNN TGCA
r5 0:0 NNN:NNN TGCA
h6 0:1 NNNN****:****NNNN

s1 0   N[14]
s2 0   *
s3 0   TNCNCGY*
s4 0   GN****
s5 0   NNN[20]
s6 0   CNRA*
```

Figure S20: Descriptor for HDV-like ribozyme (mispairs) used with RNArobo and RNAbob.

## S6.2 RNAMot descriptors

```
h1 h2 s1 h3 h4 s2 h4 h3 s3 h2 h1

h1 0:5 0
h2 4:4 0
h3 4:4 0 CNNN:NNNG
h4 0:5 0

s1 11:11 GGAAGAAACTG
s2 3:20
s3 1:1 G

M 0
W 4
```

Figure S21: Descriptor for ATP aptamer used with RNAMot.

```
h1  s4  s1  s5  h2  s6  s2  s7  h2  s8  s3  s9  h1

h1  4:6  0
h2  3:5  0

s4  0:1
s1  11:11  AAGTGGTTGGG
s5  0:1
s6  0:1
s2  4:4  UUCG
s7  0:1
s8  0:1
s3  8:8  UGUGAAAA
s9  0:1

M 5
W 10
```

Figure S22: Descriptor for GTP aptamer used with RNAMot.

```
H1  s1  H2  s2  H2  s3  H3  s4  H3  s5  H4  s6  s7  H4  H1  s8

H1  7:7  2
H2  3:4  1
H3  5:5  1
H4  5:5  1

s1  2:2  UN
s2  4:14
s3  1:1
s4  6:7
s5  2:22
s6  3:3  UUC
s7  0:4
s8  4:4  NCCA

M 3
W 20
```

Figure S23: Descriptor for tRNA used with RNAMot.

```
s1 h1 s2 h2 s3 h2 s4 h3 s5 h3 s6 h1 s7

h1 4:5 0
h2 4:5 0
h3 4:5 0

s1 15:15
s2 7:7 CTGANGA
s3 4:14
s4 4:4 GAAA
s5 4:14
s6 2:2 NH
s7 15:15

M 0
W 12
```

Figure S24: Descriptor for HHR type I used with RNAMot.

```
s1 h1 s2 h2 s3 h2 s4 h3 s5 h3 s6 h1 s7

h1 3:9 0
h2 3:9 0
h3 3:9 0

s1 15:15
s2 4:4 GAAA
s3 4:12
s4 2:2 NH
s5 4:12
s6 7:7 CTGANGA
s7 15:15

M 0
W 9
```

Figure S25: Descriptor for HHR type II used with RNAMot.

```
s1 h1 s2 h2 s3 h2 s4 h3 s5 h3 s6 h1 s7

h1 3:6 0
h2 3:6 0
h3 3:6 0

s1 10:10
s2 7:7 CTGANGA
s3 4:50
s4 4:4 GAAA
s5 4:50
s6 2:2 TN
s7 10:10

M 0
W 0
```

Figure S26: Descriptor for HHR (extended) used with RNAMot.

```
h1 h4 s1 h3 h2 s2 s7 s8 h2 h4 h1 s3 s4 s5 s9 h3 s6

h1 5:5 0 RNNNN:NNNNY
h4 0:3 0
h2 3:3 0
h3 6:8 0

s1 1:51
s2 6:6 TYYHCG
s7 0:1
s8 1:1 Y
s3 2:2 RN
s4 4:54
s5 4:4 CNRA
s9 0:1
s6 6:6

M 0
W 10
```

Figure S27: Descriptor for HDV-like ribozyme with loose P4 region used with RNAMot.

```
h1 h5 s1 h3 h2 s2 s7 s8 h2 h5 h1 s3 h4 s4 h4 s5 s9 h3 s6

h1 6:6 0 GNNNNN:NNNNNY
h5 0:1 0
h2 3:3 0
h3 6:8 0
h4 5:6 0

s1 1:51
s2 6:6 TYYHCG
s7 0:1
s8 1:1 Y
s3 2:2 RN
s4 3:6
s5 4:4 CNRA
s9 0:1
s6 6:6

M 0
W 10
```

Figure S28: Descriptor for HDV-like ribozyme with structured P4 region used with RNAMot.

```
h1 h2 s1 h3 s2 h4 h5 s3 s7 h5 h2 h1 s4 s8 h6 s5 h6 s6 s9 h3 h4

h1 1:1 0 R:Y
h2 6:6 1
h3 4:6 0
h4 3:3 0
h5 3:3 0
h6 4:8 1

s1 1:15
s2 0:1
s3 7:7 TNCNCGY
s7 0:1
s4 2:2 GN
s8 0:4
s5 3:23
s6 4:4 CNRA
s9 0:1

M 2
W 0
```

Figure S29: Descriptor for HDV-like ribozyme (mispairs) used with RNAMot.

## S6.3 RNAmotif descriptors

```
descr
  h5(tag="h1", minlen=0, maxlen=5, pair+=gu)
          h5(tag="r2", len=4)
                    ss( seq="^GGAAGAAACTG$" )
                    h5(tag="r3", len=4, seq="CNNN$")
                      h5(tag="h4", minlen=0, maxlen=5, pair+=gu)
                              ss(minlen=3, maxlen=20)
                            h3(tag="h4")
                    h3(tag="r3")
                    ss( seq="^G$" )
          h3(tag="r2")
  h3(tag="h1")
```

Figure S30: Descriptor for ATP aptamer used with RNAmotif.

```
descr
    h5(tag="h1", minlen=4, maxlen=6, pair+=gu)
        ss(minlen=0, maxlen=1)
        ss( seq="^AAGTGGTTGGG", minlen=11, maxlen=12, mismatch=2)
        h5(tag="h2", minlen=3, maxlen=5, pair+=gu)
            ss(minlen=0, maxlen=1)
            ss( seq="^UUCG", minlen=4, maxlen=5, mismatch=1)
        h3(tag="h2")
        ss(minlen=0, maxlen=1)
        ss( seq="^UGUGAAAA", minlen=8, maxlen=9, mismatch=2)
    h3(tag="h1")
```

Figure S31: Descriptor for GTP aptamer used with RNAmotif.

```
parms
          wc += gu;

descr
          h5(tag="h1", minlen=7, maxlen=7, mispair=2)
                    ss(seq="^tn$")
                    h5(tag="h2", minlen=3, maxlen=4, mispair=1)
                              ss(minlen=4, maxlen=14)
                    h3(tag="h2")
                    ss(len=1)
                    h5(tag="h3", minlen=5, maxlen=5, mispair=1)
                              ss(minlen=6, maxlen=7)
                    h3(tag="h3")
                    ss(minlen=2, maxlen=22)
                    h5(tag="h4", len=5, mispair=1)
                              ss(minlen=3, maxlen=7, seq="^ttc")
                    h3(tag="h4")
          h3(tag="h1")
          ss(seq="^ncca$")
```

Figure S32: Descriptor for tRNA used with RNAmotif.

```
descr
  ss(len=15)
  h5(tag="h1", minlen=4, maxlen=5, pair+=gu)
    ss( seq="^CTGANGA$" )
          h5(tag="h2", minlen=4, maxlen=5, pair+=gu)
                    ss( minlen=4, maxlen=14)
                  h3(tag="h2")
                  ss( seq="^GAAA$" )
                  h5(tag="h3", minlen=4, maxlen=5, pair+=gu)
                    ss( minlen=4, maxlen=14)
                  h3(tag="h3")
                  ss( seq="^NH$" )
  h3(tag="h1")
  ss(len=15)
```

Figure S33: Descriptor for HHR type I used with RNAmotif.

```
descr
  ss(len=15)
  h5(tag="h1", minlen=3, maxlen=9, pair+=gu)
    ss( seq="^GAAA$" )
          h5(tag="h2", minlen=3, maxlen=9, pair+=gu)
                ss( minlen=4, maxlen=12)
              h3(tag="h2")
              ss( seq="^NH$" )
              h5(tag="h3", minlen=3, maxlen=9, pair+=gu)
                ss( minlen=4, maxlen=12)
              h3(tag="h3")
              ss( seq="^CTGANGA$" )
  h3(tag="h1")
  ss(len=15)
```

Figure S34: Descriptor for HHR type II used with RNAmotif.

```
descr
  ss(len=10)
  h5(tag="r1", minlen=3, maxlen=6)
    ss( seq="^CTGANGA$" )
          h5(tag="r2", minlen=3, maxlen=6)
                ss( minlen=4, maxlen=50)
              h3(tag="r2")
              ss( seq="^GAAA$" )
              h5(tag="r3", minlen=3, maxlen=6)
                ss( minlen=4, maxlen=50)
              h3(tag="r3")
              ss( seq="^TN$" )
  h3(tag="r1")
  ss(len=10)
```

Figure S35: Descriptor for HHR (extended) used with RNAmotif.

```
descr
        h5(tag="h1", seq="^RNNNN", minlen=5, maxlen=8, pair+=gu)
        ss( minlen=1, maxlen=51 )

        h5(tag="h3", minlen=6, maxlen=8, pair+=gu)
            h5(tag="r4", minlen=3, maxlen=3)
                    ss(seq="^TYYHCGN\{0,1\}Y$")
            h3(tag="r4")
    h3(tag="h1")

        ss(seq="^RN$")
        ss( minlen=4, maxlen=54 )
        ss( minlen=4, maxlen=5, seq="^CNRA")

        h3(tag="h3")
        ss( minlen=6, maxlen=6 )
```

Figure S36: Descriptor for HDV-like ribozyme with loose P4 region used with RNAmotif.

```
descr
        h5(tag="h1", seq="^GNNNN", minlen=6, maxlen=7, pair+=gu)
        ss( minlen=1, maxlen=51 )

        h5(tag="h3", minlen=6, maxlen=8, pair+=gu)
            h5(tag="r4", minlen=3, maxlen=3)
                    ss(seq="^TYYHCGN\{0,1\}Y$")
            h3(tag="r4")
    h3(tag="h1")

        ss(seq="^RN$")
        h5(tag="h4", minlen=5, maxlen=6, pair+=gu)
            ss( minlen=3, maxlen=6 )
    h3(tag="h4")
        ss( minlen=4, maxlen=5, seq="^CNRA")
        h3(tag="h3")
        ss( minlen=6, maxlen=6 )
```

Figure S37: Descriptor for HDV-like ribozyme with structured P4 region used with RNAmotif.

```
descr
        h5(tag="h1", seq="^R$", pair+=gu)
        h5(tag="r2", len=6, mispair=1)
                ss( minlen=1, maxlen=15 )

                h5(tag="r3", minlen=4, maxlen=6)
                  ss(minlen=0, maxlen=1)

                h5(tag="r4", minlen=3, maxlen=3)

                        h5(tag="r5", minlen=3,maxlen=3)
                                ss(seq="^TNCNCGY",  minlen=7, maxlen=8)
                        h3(tag="r5")
        h3(tag="r2")
  h3(tag="h1")

        ss( minlen=2, maxlen=6, seq="^GN")

        h5(tag="h6", minlen=4, maxlen=8, mispair=1, pair+=gu)
          ss( minlen=3, maxlen=23 )
        h3(tag="h6")

        ss( minlen=4, maxlen=5, seq="^CNRA")

        h3(tag="r3")
    h3(tag="r4")
```

Figure S38: Descriptor for HDV-like ribozyme (mispairs) used with RNAmotif.

# S7 Comparison with RaligNAtor

In this section, we provide additional details of the experimental comparison of RNArobo running times with RaligNAtor, a recent tool by Meyer *et al.* (2013). RaligNAtor provides two main search methods, *lscan* for online search, and *lgslink* that uses an index built in advance for the sequence database.

RaligNAtor can search for structural patterns, specified as a set of non-overlapping substructures that do not share any elements (for example a hairpin cannot be split to substructures). In each such substructure, users can allow indels, replacements, or mis-pairs, each with an individual penalty cost. However pseudo-knotted structures are not possible.

We compared the running time on two structural motifs: Cripavirus internal ribosome entry site (IRES) contained in the RaligNAtor package, and the generalized tRNA motif from our experiments. Additionally, we conducted search for the IRES motif with no distortions allowed. We searched the motifs in the sequences from the Rfam 11 database, and in the whole genome of *Drosophila melanogaster*.

The structural pattern definition language used by RaligNAtor (Meyer *et al.*, 2013) is not compatible with RNArobo, as RaligNAtor enables to set distortion parameters only for whole substructures, while RNArobo uses element-wise parametrization. Additionally, RNArobo does not support user-defined penalty cost schemes. To compare the running time, we approximately translated the motif description from RaligNAtor format to RNArobo format in case of IRES, and the other way around for tRNA (see descriptors in Figures S39, S40, S41, and S42). For both motifs, the RNArobo descriptor yields more unique hits in the searched sequences (see Table S1). Since matches generally slow down RNArobo search, the differences in the descriptors should not favor RNArobo.

The running times are presented in Table S2. RNArobo outperformed both RaligNAtor's online and index search methods. Building index for *lgslink* took 13.4 and 58 seconds for Rfam 11 and genome of *D. melanogaster*, respectively, while increasing the storage requirements by 7.3 and 11.3 times, respectively.

Table S1: The number of unique matches found in the comparison of RNArobo and RaligNAtor.

| | Rfam11 | | | *D. melanogaster* genome | | |
|---|---|---|---|---|---|---|
| | RNArobo | *lgslink* | *lscan* | RNArobo | *lgslink* | *lscan* |
| IRES | 8 | 4 | 4 | 0 | 0 | 0 |
| IRES (exact) | 0 | 0 | 0 | 0 | 0 | 0 |
| tRNA | 1986 | 914 | 914 | 7570 | 207 | 207 |

Table S2: The running time of RNArobo and RaligNAtor (in seconds) when searched on both positive and negative strands.

| | Rfam11 | | | *D. melanogaster* genome | | |
|---|---|---|---|---|---|---|
| | RNArobo | *lgslink* | *lscan* | RNArobo | *lgslink* | *lscan* |
| IRES | **35.75** | 3139.71 | 4107.73 | **3433.89** | 15888.38 | 15404.81 |
| IRES (exact) | **3.65** | 21.38 | 448.28 | **5.24** | 114.77 | 1884.98 |
| tRNA | **6.41** | 48543.91 | 10014.62 | **13.82** | 246782.23 | 51288.21 |

```
>ires1|cost=2|indels=0
UGAWCUKD
........
>ires2|indels=1|cost=4
DNNNDNDNHNDMWWDYBVNVDNBWHDWADNNNNNNH
((((((........................))))))
>ires3|indels=0|cost=1
VNHUAUUUADNBWUAC
((((....))))....
>ires4|indels=2|cost=3
CARGAYSNVNNNNDGCRKYCCHVHRWNRUCYAG
(.(((((....((((.....))))..))))).)
>ires5|indels=1|cost=3|deletion=2
BHKHDHDSNBHDRGUNSNSNNNWNN
(((...((((......))))..)))
```

Figure S39: RaligNAtor structural pattern for Cripavirus internal ribosome entry site (IRES) as listed in RaligNAtor 1.2 manual (Meyer *et al.*, 2013).

```
s1 sX1  h1 s2 h1' sX2  h2 s3 h2' s4 sX3  h3 s5 h4 s6 h5 s7 h5' s8 h4' s9 h3' sX4
h6 s10 h7 s11 h7' s12 h6'

s1 2 UGAWCUKD
sX1 0 [30]
h1 1:2:1 DNNNDN:NNNNNH:N
s2 2:1 DNHNDMWWDYBVNVDNBWHDWADN:N
sX2 0 [30]
h2 1:1 VNHU:ADNB
s3 1 AUUU
s4 1 WUAC
sX3 0 [30]
h3 0:0 C:G
s5 0 A
h4 1:2:1 RGAYS:NRUCY:N
s6 0:1 NVNN:N
h5 1:2:1 NNDG:CHVH:N
s7 1:1 CRKYC:N
s8 0 RW
s9 0 A
sX4 0 [30]
h6 1:2 BHK:WNN
s10 0:1 HDH:N
h7 1:2 DSNB:SNSN
s11 0:1 HDRGUN:N
s12 0 NN
```

Figure S40: Descriptor for IRES used with RNArobo.

```
>tRNA-pat|indels=5|cost=5|replacement=100
NNNNNNNTNNNNNNNNNNNNNNNNNNNNNNNNNNNNNNNNNNNNNNNNNNNNNNNNNNTTCNNNNNNNNNNNNNNNNNCCA
(((((((..((((........)))).(((((......))))).........(((((.......))))))))))))....
```

Figure S41: Structural pattern of tRNA as used for search with RaligNAtor. Only up to 5 indels in the whole pattern are admissible, as cost of single replacement is set above overall penalty allowance.

```
h1 s1 h2 s2 h2' s3 h3 s4 h3' s5 h4 s6 h4' h1' s8

h1 0:2 NNNNNNN:NNNNNNN
h2 0:1 *NNN:NNN*
h3 0:1 NNNNN:NNNNN
h4 0:1 NNNNN:NNNNN
s1 0 TN
s2 0 NNNN**********
s3 0 N
s4 0 NNNNNN*
s5 0 NN********************
s6 0 TTC****
s8 0 NCCA
```

Figure S42: Descriptor for tRNA used with RNArobo (Eddy, 1996).

# References


Carothers, J. M., Davis, J. H., Chou, J. J., and Szostak, J. W. (2006). Solution structure of an informationally complex high-affinity RNA aptamer to GTP. *RNA*, **12**(4), 567–579.

Davis, J. H. and Szostak, J. W. (2002). Isolation of high-affinity GTP aptamers from partially structured RNA libraries. *Proceedings of the National Academy of Sciences*, **99**(18), 11616–11621.

Eddy, S. (1996). RNABob: a program to search for RNA secondary structure motifs in sequence databases. unpublished.

Gautheret, D., Major, F., and Cedergren, R. (1990). Pattern searching/alignment with RNA primary and secondary structures: an effective descriptor for tRNA. *Comput Appl Biosci*, **6**(4), 325–31.

Holt, R. A., Subramanian, G. M., Halpern, A., Sutton, G. G., Charlab, R., Nusskern, D. R., Wincker, P., Clark, A. G., Ribeiro, J. C., Wides, R., *et al.* (2002). The genome sequence of the malaria mosquito Anopheles gambiae. *Science*, **298**(5591), 129–149.

Ivanova, N., Sorokin, A., Anderson, I., Galleron, N., Candelon, B., Kapatral, V., Bhattacharyya, A., Reznik, G., Mikhailova, N., Lapidus, A., *et al.* (2003). Genome sequence of Bacillus cereus and comparative analysis with Bacillus anthracis. *Nature*, **423**(6935), 87–91.

Jimenez, R., Rampášek, L., Brejová, B., Vinař, T., and Lupták, A. (2012). Discovery of RNA motifs using a computational pipeline that allows insertions in paired regions and filtering of candidate sequences. *Methods in molecular biology (Clifton, NJ)*, **848**, 145.

Lorenz, R., Bernhart, S., zu Siederdissen, C., Tafer, H., Flamm, C., Stadler, P., and Hofacker, I. (2011). ViennaRNA package 2.0. *Algorithms for Molecular Biology*, **6**(1), 26.

Meyer, F., Kurtz, S., and Beckstette, M. (2013). Fast online and index-based algorithms for approximate search of rna sequence-structure patterns. *BMC bioinformatics*, **14**(1), 226.

Perreault, J., Weinberg, Z., Roth, A., Popescu, O., Chartrand, P., Ferbeyre, G., and Breaker, R. R. (2011). Identification of hammerhead ribozymes in all domains of life reveals novel structural variations. *PLoS Comput Biol*, **7**(5), e1002031.

Ruminski, D. J., Webb, C.-H. T., Riccitelli, N. J., and Lupták, A. (2011). Processing and translation initiation of non-long terminal repeat retrotransposons by hepatitis delta virus (HDV)-like self-cleaving ribozymes. *Journal of Biological Chemistry*, **286**(48), 41286–41295.

Sodergren, E., Weinstock, G. M., Davidson, E. H., Cameron, R. A., Gibbs, R. A., Angerer, R. C., Angerer, L. M., Arnone, M. I., Burgess, D. R., Burke, R. D., *et al.* (2006). The genome of the sea urchin Strongylocentrotus purpuratus. *Science*, **314**(5801), 941–952.

Sperschneider, J. and Datta, A. (2010). DotKnot: pseudoknot prediction using the probability dot plot under a refined energy model. *Nucleic acids research*, **38**(7), e103–e103.

Webb, C. H., Riccitelli, N. J., Ruminski, D. J., and Luptak, A. (2009). Widespread occurrence of self-cleaving ribozymes. *Science*, **326**(5955), 953.

Webb, C.-H. T. and Lupták, A. (2011). HDV-like self-cleaving ribozymes. *RNA biology*, **8**(5), 719–727.
